# Immunomodulatory function of the cystic fibrosis modifier gene *BPIFA1*

**DOI:** 10.1371/journal.pone.0227067

**Published:** 2020-01-13

**Authors:** Aabida Saferali, Anthony C. Tang, Lisa J. Strug, Bradley S. Quon, James Zlosnik, Andrew J. Sandford, Stuart E. Turvey

**Affiliations:** 1 Centre for Heart Lung Innovation, University of British Columbia and St Paul’s Hospital, Vancouver, British Columbia, Canada; 2 Department of Pediatrics, University of British Columbia and BC Children’s Hospital, Vancouver, British Columbia, Canada; 3 Channing Division of Network Medicine, Brigham and Women’s Hospital, Boston, Massachusetts, United States of America; 4 Harvard Medical School, Boston, Massachusetts, United States of America; 5 Program in Genetics and Genome Biology, The Hospital for Sick Children, Division of Biostatistics, Dalla Lana School of Public Health, University of Toronto, Toronto, Ontario, Canada; University of Pittsburgh, UNITED STATES

## Abstract

**Background:**

Cystic fibrosis (CF) is characterized by a progressive decline in lung function due to airway obstruction, infection, and inflammation. CF patients are particularly susceptible to respiratory infection by a variety of pathogens, and the inflammatory response in CF is dysregulated and prolonged. BPI fold containing family A, member 1 (BPIFA1) and BPIFB1 are proteins expressed in the upper airways that may have innate immune activity. We previously identified polymorphisms in the *BPIFA1/BPIFB1* region associated with CF lung disease severity.

**Methods:**

We evaluated whether the *BPIFA1/BPIFB1* associations with lung disease severity replicated in individuals with CF participating in the International CF Gene Modifier Consortium (n = 6,365). Furthermore, we investigated mechanisms by which the BPIFA1 and BPIFB1 proteins may modify lung disease in CF.

**Results:**

The association of the G allele of rs1078761 with reduced lung function was replicated in an independent cohort of CF patients (p = 0.001, n = 2,921) and in a meta-analysis of the full consortium (p = 2.39x10^-5^, n = 6,365). Furthermore, we found that rs1078761G which is associated with reduced lung function was also associated with reduced BPIFA1, but not BPIFB1, protein levels in saliva from CF patients. Functional assays indicated that BPIFA1 and BPIFB1 do not have an anti-bacterial role against *P*. *aeruginosa* but may have an immunomodulatory function in CF airway epithelial cells. Gene expression profiling using RNAseq identified Rho GTPase signaling pathways to be altered in CF airway epithelial cells in response to treatment with recombinant BPIFA1 and BPIFB1 proteins.

**Conclusions:**

BPIFA1 and BPIFB1 have immunomodulatory activity and genetic variation associated with low levels of these proteins may increase CF lung disease severity.

## Introduction

The upper respiratory tract, starting with the nasal and oral cavities, is a major route for entry of pathogens into the body. As well as serving as a structural barrier, airway epithelial cells produce proteins that are secreted into the airway lumen and provide a first line of defense against pathogenic exposures. Some of the most highly expressed proteins in the upper airways are members of the BPI fold (BPIF) family, including BPIFA1 (SPLUNC1, short palate lung nasal epithelium clone 1) and BPIFB1 (LPLUNC1, lung palate lung nasal epithelium clone 1), that are secreted by airway epithelial cells [1]. Protein levels of both BPIFA1 and BPIFB1 have been shown to be upregulated in subjects with cystic fibrosis (CF) [2, 3], suggesting that these molecules may have a role in the disease.

We previously demonstrated that genetic variants in the *BPIFA1/BPIFB1* region are associated with decreased gene expression and increased lung disease severity in cystic fibrosis (CF) [4]. This suggests that decreased *BPIFA1* and/or *BPIFB1* expression may be detrimental to CF lung function. Several recent studies have further confirmed that *BPIFA1* variants can contribute to disease by altering protein levels or function. A study of *BPIFA1* in asthma demonstrated that the CC genotype of the rs750064 polymorphism is associated with reduced BPIFA1 expression in asthmatic nasal epithelial cells, and higher proinflammatory response to IL-13 treatment [5]. Recently, a rare missense variant in *BPIFA1* was identified in patients with meningococcal disease and was found to reduce antibiofilm activity, meningococcal adhesion, and invasion of cells [6].

There are several potential mechanisms by which BPIFA1 and BPIFB1 could modulate CF disease severity. BPIFA1 has been shown to inhibit the growth of several bacterial species [7–10], as well as to bind lipopolysaccharide [11]. Transgenic mice overexpressing human BPIFA1 have enhanced bacterial clearance of *P*. *aeruginosa*, together with reduced inflammatory cytokine production [12]. *BPIFA1* knockout mice have impaired bacterial clearance and increased levels of inflammatory cells [9, 10, 13, 14]. Furthermore a BPIFA1 peptide has been shown to restrict influenza A virus infection. [15].

BPIFA1 also has immunomodulatory functions in mouse models of airway inflammation. Mice that are deficient in Bpifa1 have higher levels of eosinophils, mucus production, airway hyper-reactivity, interleukin (IL)-4, IL-5, and IL-13 [16, 17]. In contrast, BPIFA1 has pro-inflammatory properties, as mice overexpressing human BPIFA1 produced elevated levels of TNF-α and IL-6 in response to stimulation with carbon nanotubes [18]. Furthermore, BPIFA1 functioned as a chemoattractant *in vitro* by enhancing neutrophil migration [7]. BPIFA1 may also contribute to CF by modulating the function of the Epithelial Sodium Channel (ENaC), which is dysregulated in the disease [19], resulting in reduced Na^+^ and water transport movement across the airway epithelium[20, 21].

Evidence that BPIFB1 plays a role in innate immunity comes from a genetic association with clinical outcomes in cholera [22] in combination with data indicating that BPIFB1 modifies the innate immune response to *Vibrio cholera* [23].

In this study, we build upon our previous work by using an independent CF cohort to replicate the observation that genetic variation in the *BPIFA1/BPIFB1* region is associated with CF lung disease severity. Moreover, we provide insight into the causal variant and gene responsible for this robust and replicated association, and we establish that BPIFA1 and BPIFB1 have immunomodulatory functions that may modify lung disease in CF.

## Methods

### Association of rs1078761 with CF lung disease severity

To assess replication of the association of rs1078761 with CF lung disease severity, data from a published GWAS for lung disease severity in CF were interrogated [24]. Briefly, genotyping was performed using the Illumina 660W, Omni5, CNV370 or 610 platforms. Imputation was performed using MaCH/Minimac software and the 1000 Genomes Phase I, Version 3 reference population. Lung disease severity was quantified using the previously described KNoRMA phenotype which represents multiple measurements of CF-specific forced expiratory volume at one second (FEV_1_) percentiles adjusted for age, sex and mortality [25].

### Recruitment of CF patients and sample processing

CF patients were recruited from the adult CF clinic at the Pacific Lung Health Centre, St. Paul’s Hospital, Vancouver, Canada. Subjects were recruited if they had a confirmed diagnosis of CF based on sweat chloride testing and/or genotyping. Saliva samples were collected from 30 CF patients during a routine stable clinic visit. Patients were asked to rinse their mouths with bottled water and to spit into a sterile collection container 5 times over 5 minutes. Saliva samples were processed within one hour of collection by adding Sputolysin reagent (Calbiochem, San Diego, CA, USA) to a ratio of 4 mL Sputolysin per 1 gram of saliva. Samples were then incubated in a 37°C water bath for 20 minutes with shaking by inversion every 5 minutes. Samples were centrifuged at 500 relative centrifugal force (RCF) for 10 minutes at 4° C, and the supernatant was again centrifuged at 4000 RCF for 20 minutes at 4° C. Samples were aliquoted and frozen at -80°C until analysis. Pellets from saliva centrifugation were stored for DNA extraction.

### Immunoblot analysis of BPIFA1 and BPIFB1 protein levels in saliva

Total protein was quantified in saliva samples using the Coomassie plus (Bradford) assay (Sigma-Aldrich, Oakville, Ontario, Canada). 11.2 μg of total protein was run on 12% polyacrylamide gels which were probed for BPIFA1 and BPIFB1 using a goat anti-human BPIFA1 antibody (R&D Systems, Minneapolis, MN, USA) and a mouse anti-human BPIFB1 antibody (Sigma-Aldrich). Donkey anti-goat (Life Technologies, Burlington, ON, Canada) and goat anti-mouse (Rockland Immunochemicals, Gilbertsville, PA, USA) fluorescently conjugated antibodies were used for detection. A reference sample was run on each gel to normalize for differences between gels. Densitometry was performed using Image J to measure the intensity of the BPIFA1 and BPIFB1 signals.

### DNA extraction and genotyping of saliva samples

DNA was extracted from saliva pellets using the QIAamp DNA Mini Kit (Qiagen, Valencia CA, USA). 5 ng of DNA was genotyped using TaqMan assays (Life Technologies) for rs1078761. Genotyping was performed on the Applied Biosystems ViiA7 Real-Time PCR System (Life Technologies). DNA samples from CEPH individuals of known genotype were used as positive controls (Coriell Institute, Camden, New Jersey, USA).

### Bacterial growth assays

Bacterial growth assays were performed using the PAO1 strain of *P*. *aeruginosa*, which is a common laboratory strain obtained from a wound isolate. Frozen bacterial cultures were streaked out onto LB agar plates and incubated overnight at 37°C. Individual colonies were selected and grown overnight in 3 mL of LB broth in a 37°C incubator with shaking. The optical density of the overnight cultures was measured and cultures were diluted to an optical density of 0.05. Cultures were pipetted into the wells of a honeycomb microplate (Growth Curves USA, USA) and cultures were treated with 1 or 10 μg of recombinant BPIFA1 (Abnova, Walnut, CA, USA), or PBS as a negative control. Each condition was performed in triplicate, and each experiment was performed at least 3 times. The microplates were incubated in the Bioscreen C (Growth Curves USA, USA), an automated microbiology growth curve analysis system which was used to incubate cultures at 37°C with shaking, with measurements of optical density performed every 15 minutes. After 24 hours of growth, cultures were serially diluted and two to three dilutions were plated onto agar plates and incubated at 37°C overnight. Colonies were counted after overnight growth to quantify colony forming units for each treatment condition.

### Cell culture

IB3-1 and CuFi-1 CF airway epithelial cell lines were obtained from the American Type Culture Collection (Manassas, VA, USA). CFBE41o- cells were generously shared from the laboratory of Dr Dieter Grunert (University of California, San Francisco, CA, USA). IB3-1 cells were derived from a patient who was a compound heterozygote for the p.Phe508del and W1282X CFTR mutations, and CuFi-1 and CFBE41o- cells were derived from p.Phe508del homozygous patients. Cells were cultured as recommended by ATCC using standard protocols. IB3-1 cells were grown in basal LHC-8 medium (Thermo Fisher Scientific, Waltham, MA, USA) supplemented with 10% fetal bovine serum (FBS), 2mM L-glutamine, and 1mM sodium pyruvate. CuFi-1 cells were cultured in BEBM serum-free medium (Lonza, Anaheim, CA, USA) with supplement bullet kit (EGF, hydrocortisone, bovine pituitary extract, transferrin, bovine insulin, triiodothyronine, epinephrine, retinoic acid), 2 mM L-glutamine, and 1mM sodium pyruvate. CFBE41o- cells were grown in minimum essential medium with Earle’s salt (Sigma-Aldrich) supplemented with 10% FBS and Glutamax (Thermo Fisher Scientific). Prior to stimulation, airway epithelial cells were plated in coated 24-well plates (BD Biosciences, San Jose, CA, USA) at 1.7×10^5^ cells/well, and allowed to adhere overnight. Plates used for IB3-1 and CFBE41o- stimulations were precoated in a mixture of bovine serum albumin (10 mg/cm^2^), fibronectin (1 mg/cm^2^), and bovine collagen type I (3.3 mg/ cm^2^) (BD Biosciences). Plates for CuFi-1 were precoated with collagen type IV (6 mg/ cm^2^) (Sigma-Aldrich).

### BPIFA1 overexpression assays

IB3-1 cells were seeded in 12-well plates and allowed to achieve ~80% density over 1 to 3 days. A human cDNA clone of the complete open reading frame of transcript variant 1 of *BPIFA1* (RefSeq NM_016583.2) was purchased from Origene (Origene, Rockville, MD, USA). Cells were transfected with 500 ng of *BPIFA1* total plasmid or empty pCMV6-entry vector (Origene) using Lipofectamine 2000 (Thermo Fisher Scientific) according to the manufacturer’s instructions. After transfection overnight, the medium was replaced with 350 μl of fresh LHC-8 medium (Thermo Fisher Scientific) and the cells were allowed to rest overnight. *P*. *aeruginosa* (PAO1 strain) was grown from an overnight culture to log phase (~ 2 hours) and used to infect cultures at a MOI of 10 (confluent 12-well plate of IB3-1 ~ 180,000 cells). After 5 hours of incubation, a portion of the supernatant was obtained for colony forming unit (CFU) counts, and the rest was clarified by centrifugation for ELISA and immunoblotting. Cells were also collected to make lysates. For CFU quantification, supernatants were serially diluted 10-fold, plated on LB plates, and allowed to incubate overnight at 37°C prior to counting the next day. To confirm that the transfection had been successful, supernatants were immunoblotted for the presence of BPIFA1 protein. In order to control for the potential inflammatory background associated with nucleic acid transfection, the amount of plasmid for each condition was normalized to 500 ng per transfection. The conditions were as follows: 500 ng BPIFA1 plasmid, 100 ng *BPIFA1* plasmid + 400 ng pCMV6 vector, and 500 ng pCMV6 vector.

### Cell stimulation and cytokine quantification

Airway epithelial cells were pretreated for 30 minutes with 5 μg of recombinant BPIFA1 (Abnova), recombinant BPIFB1 (Abnova), or fresh media as a negative control. Heat killed *P*. *aeruginosa* (using the PAO1 strain) were generated by incubating bacterial cultures at 95°C for 30 min. Pretreated and untreated airway epithelial cells were stimulated with heat killed *P*. *aeruginosa* at a multiplicity of infection (MOI) of 50. Cell lysates were collected at 8 hours after stimulation for extraction of RNA, and supernatants were collected after 24 hours for measurement of cytokine levels (unless otherwise specified). IL-6 and IL-8 levels were quantified in cell supernatants using sandwich ELISA (Thermo Fisher Scientific), according to the manufacturer’s instructions.

### RNA extraction and sequencing

RNA was extracted from cell lysates using the RNeasy Mini kit (Qiagen). RNA concentration, integrity and purity were assessed using the RNA Nano Kit with the Agilent 2100 Bioanalyzer (Agilent Technologies, Santa Clara, CA, USA). mRNA was reverse transcribed, amplified and sequenced using Ion Torrent library kits and the Ion Proton next generation sequencing system (Thermo Fisher Scientific) at the University of British Columbia sequencing core, Djavad Mowafaghian Centre for Brain Health, Vancouver, BC, Canada. All samples were sequenced to a depth of at least 15 million reads. Sequencing quality was assessed using FastQC [26], and adapter sequences were trimmed from reads using cutadapt [27]. Reads were mapped to the human genome (hg19) with a two-step alignment protocol using Tophat2 followed by Bowtie2 [28, 29]. Sorting and indexing of BAM and SAM files was performed using SAMtools [30]. HTSeq-count was used to generate read count tables. Differential gene expression analysis was performed using the Bioconductor packages DESeq2 [31] and Limma [32]. A fold change threshold greater than or equal to ± 1.5 was used to identify differentially expressed genes. Downstream analysis was performed using Sigora [33] for pathway analysis, and NetworkAnalyst to generate protein:protein interaction networks for differentially expressed genes (http://www.networkanalyst.ca/NetworkAnalyst/)[34].

## Results

### rs1078761 is a replicated modifier of CF lung disease severity

**Fig 1:**
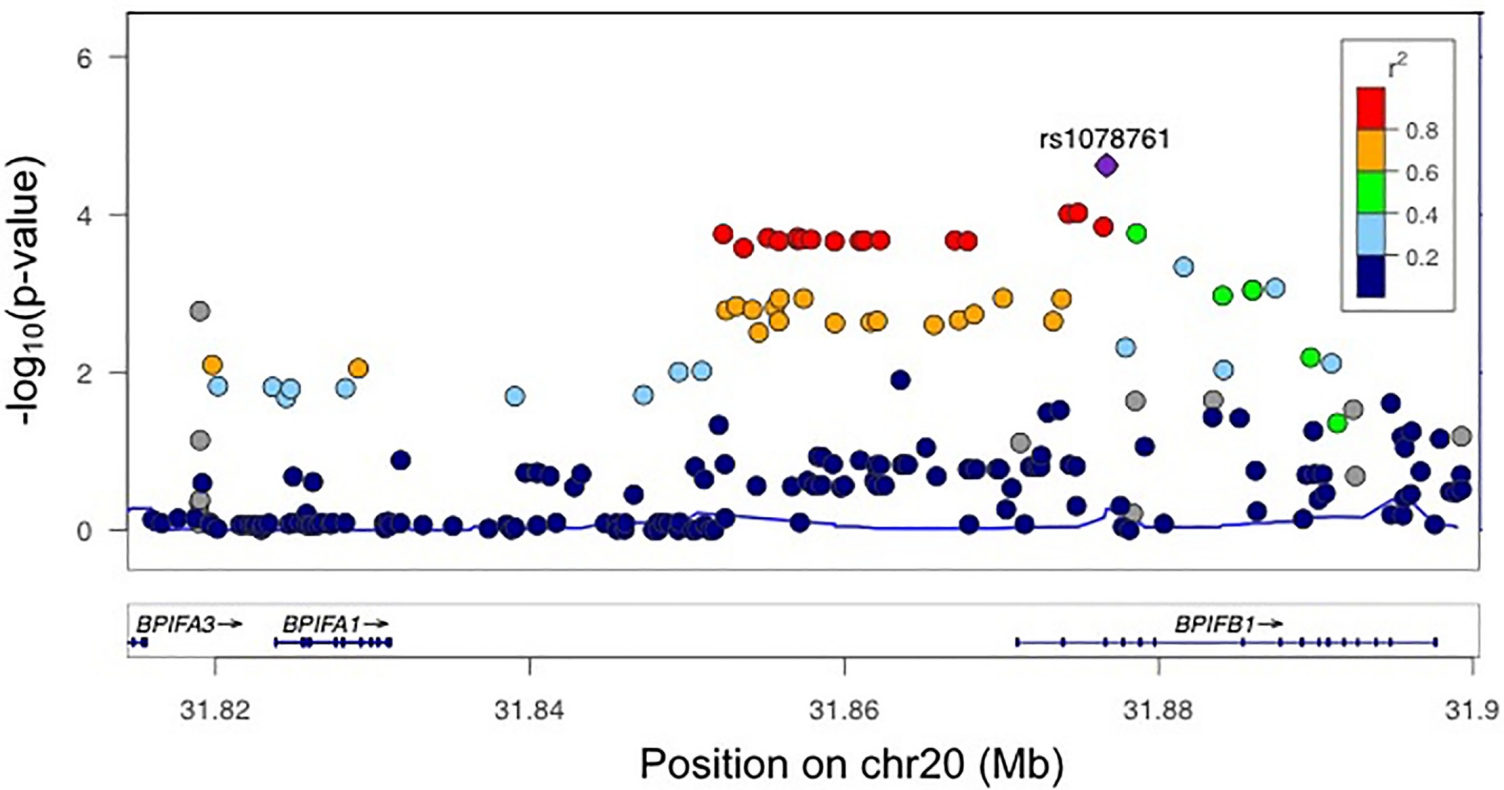
Locus zoom plot of the region 10 kb upstream of *BPIFA1* to 10 kb downstream of *BPIFB1*. The *y* axis shows *P* values in the–log_10_ scale for polymorphisms that were tested for association in the meta-analysis of 6365 CF patients for lung disease severity. rs1078761 remained the most significant association in the region (purple diamond). The extent of linkage disequilibrium (measured by the r^2^ statistic) with rs1078761 for the remaining SNPs is indicated with colors.

To test for replication of our previous association [4] of rs1078761 with CF lung disease severity, we assessed an independent cohort of 2,921 CF individuals (Tables 1 and 2). In the replication group, the G allele of rs1078761 was significantly associated with decreased CF lung function (*P* = 0.001), replicating our previous findings [4]. In a meta-analysis of all individuals from the initial and replication cohorts incorporating 6,365 CF patients from the International CF Gene Modifier Consortium, the *P* value for rs1078761 was 2.39×10^−5^. The rs1078761 polymorphism remained the most significant polymorphism in the region (Fig 1).

**Table 1:** Characteristics of the individuals included in the replication and meta-analysis of rs1078761.

	Study	Lead Institution	Design	Number of Subjects	Total
**Replication cohort**	French CF Gene Modifier Consortium	Université of Pierre and Marie Curie	Population-based	1222	2333
	Genetic Modifier Study	University of North Carolina	Extremes of phenotype	469	
			Not Extremes of phenotype	357	
	Canadian Consortium for Genetic Studies	Hospital for Sick Children	Population-based	285	
**Meta-analysis of Replication and Initial Cohort**					6365

**Table 2:** Results for genetic association of rs1078761 with CF lung disease severity.

	Reference/ Alternative Allele^1^	p-value (fixed effects)^2^	p-value (random effects)^3^	beta score (fixed effects)^4^	beta score (random effects)
Replication cohort	G/A	0.001	0.0069	-0.075	-0.069
Meta -analysis of Replication and Initial Cohort	G/A	2.39 x 10^−5^	0.0033	-0.068	-0.063

^1^Reference allele refers to the allele used when calculating the beta score

^2^The fixed-effects method for meta-analysis assumes that the true effect of the risk allele is the same in each data set

^3^The random effects method for meta-analysis models heterogeneity between studies

^4^The beta-score provides information about the effect size and direction of effect. In this case, a negative beta score indicates that the reference allele is associated with reduced lung function

### The rs1078761G risk genotype is associated with lower levels of BPIFA1 protein in saliva from clinically stable CF patients

**Fig 2:**
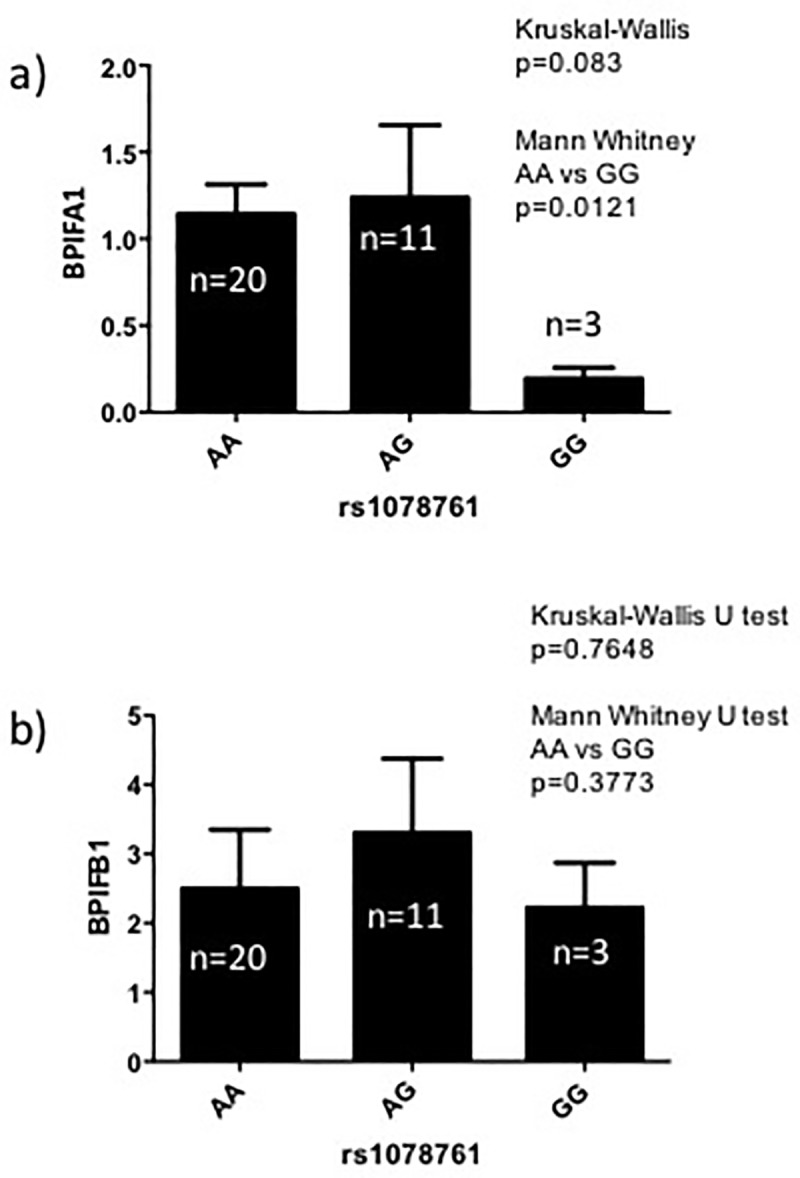
Association between rs1078761 genotype and BPIFA1 levels in the saliva of clinically stable patients with CF. The *y* axis shows *P* values in the–log_10_ scale for polymorphisms that were tested for association in the meta-analysis of 6365 CF patients for lung disease severity. rs1078761 remained the most significant association in the region (purple diamond). The extent of linkage disequilibrium (measured by the r^2^ statistic) with rs1078761 for the remaining SNPs is indicated with colors.

To understand how *BPIFA1* genotype may modify lung disease severity in patients living with CF, we quantified the BPIFA1 protein levels in the saliva of CF patients who were all clinically stable. CF patients who were homozygous for the G allele, which is associated with worse lung function, had significantly reduced levels of BPIFA1 protein compared to AA homozygotes (*P* = 0.012) (Fig 2A). BPIFB1 levels were not different between genotypes (Fig 2B), suggesting that decreased BPIFA1 may be responsible for the association between rs1078761 genotype and CF lung disease severity.

### Investigation of bacterial growth inhibition by BPIFA1 and BPIFB1

**Fig 3:**
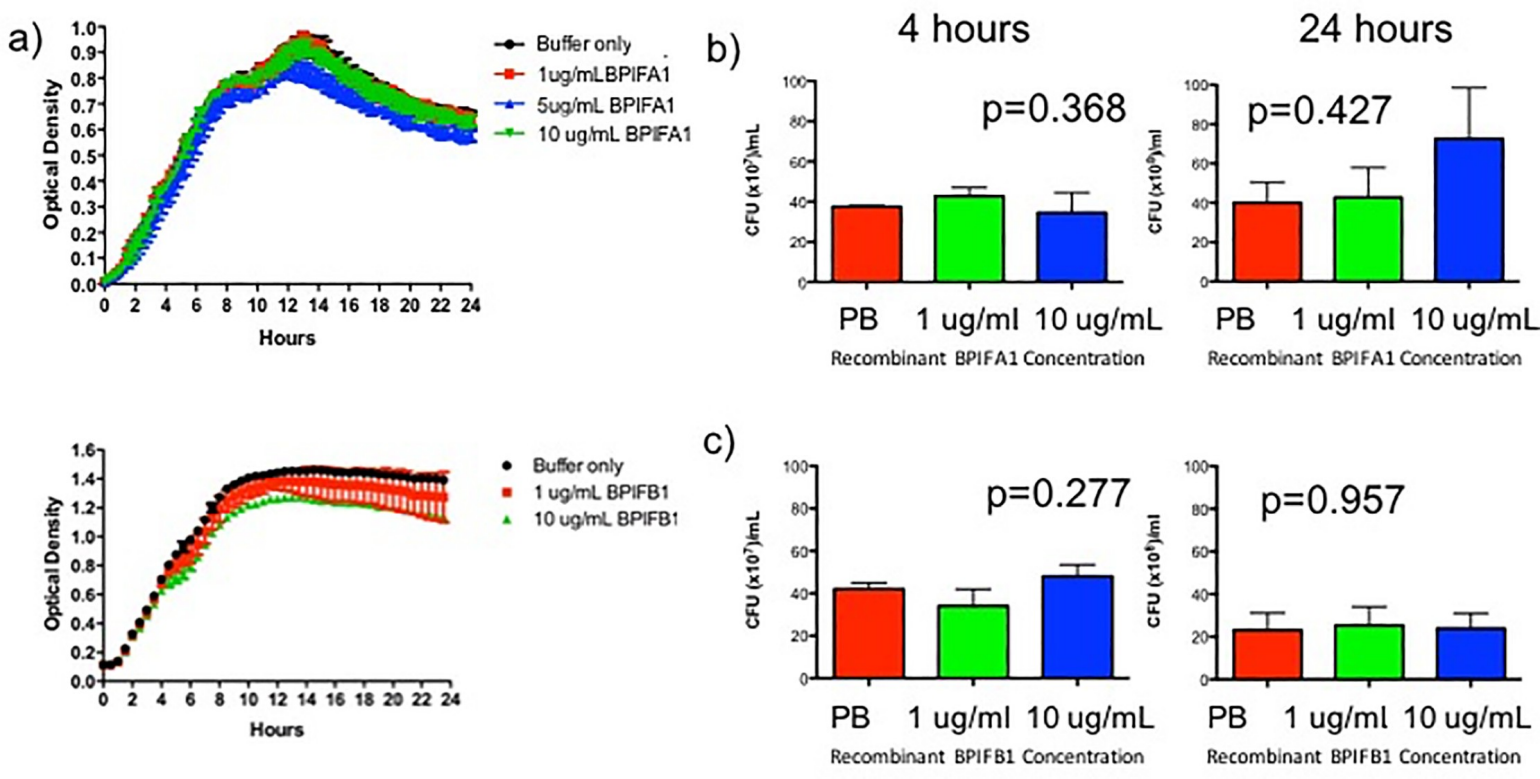
*P*. *aeruginosa* growth curves and colony forming units with the addition of BPIFA1 or BPIFB1 recombinant proteins. a) Growth curves of the PAO1 strain of *P*. *aeruginosa* treated with 1, 5 and 10 μg of recombinant BPIFA1 or 1 and 10 μg of BPIFB1 protein. b-c) Quantification of colony forming units in *P*. *aeruginosa* pretreated with recombinant BPIFA1 (b) or recombinant BPIFB1 (c). Values represent mean plus standard error of the mean. The Kruskal-Wallis test was used to assess for statistical differences in colony forming units between conditions and the corresponding *P* values are shown. Colony forming units were measured at 4 hours and 24 hours of bacterial culture growth.

Published data suggest that BPIFA1 inhibits the growth of some bacteria [7–10, 13, 14, 35]. Therefore, to better understand how genetic variation in this locus may modify the severity of CF lung disease, we directly assessed whether BPIFA1 and BPIFB1 inhibited growth of *P*. *aeruginosa*—the most clinically relevant CF pathogen. We incubated bacterial cultures with recombinant BPIFA1 or BPIFB1 protein. Experiments were designed to assess both the bactericidal and bacteriostatic effects of the recombinant proteins by combining analysis of growth curves with quantification of colony forming units (CFUs). There were no changes in the growth curves with the addition of BPIFA1 or BPIFB1 (Fig 3A) and there were no changes in CFU counts with the addition of BPIFA1 or BPIFB1 (*P* = 0.427 and 0.957 respectively, at 24 hours of bacterial growth) (Fig 3B and 3C). Together, these data indicate that BPIFA1/2 do not directly inhibit the growth the PAO1 strain of *P*. *aeruginosa*.

### Effect of BPIFA1 over-expression on bacterial growth and inflammatory cytokine production

**Fig 4:**
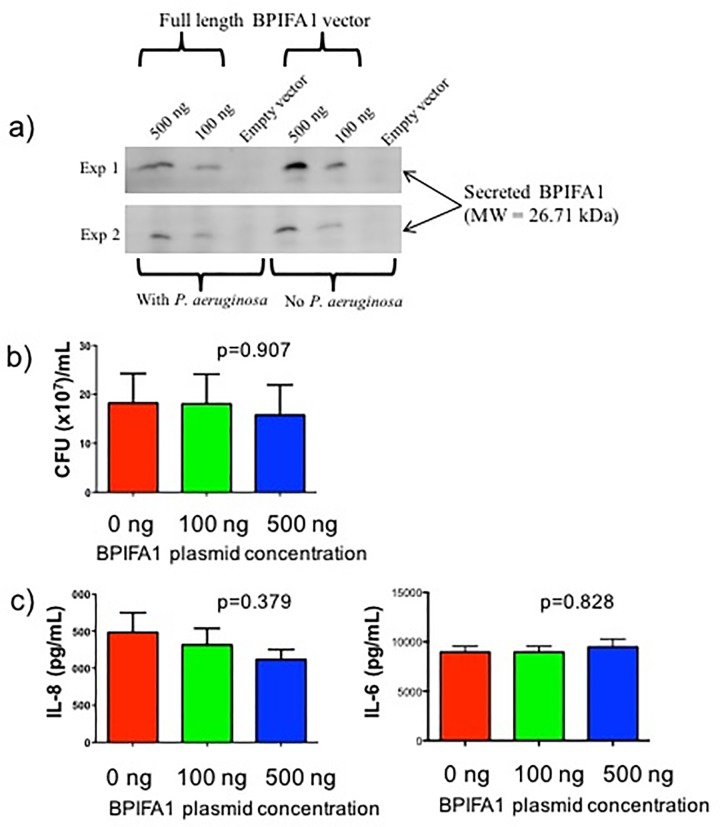
Quantification of colony forming units and inflammatory cytokine production in IB3-1 cells transfected with BPIFA1 plasmid. IB3-1 cells were transfected with empty vector, 100 ng or 500 ng of BPIFA1 plasmid, and stimulated with *P*. *aeruginosa* for 5 hours. This was followed by a) detection of secreted BPIFA1 in transfected IB3-1 cells, b) quantification of colony forming units, and c) quantification of IL-8 and IL-6 production by ELISA. Values represent mean plus standard error of the mean. The Kruskal-Wallis test was used to determine statistical differences between conditions and the corresponding *P* values are shown.

To understand the cell context-dependent role of BPIFA1, we next tested whether BPIFA1 produced by human CF airway epithelial cells inhibited bacterial growth *in vitro*. IB3-1 cells did not secrete detectable amounts of BPIFA1 protein at baseline as assessed by immunoblot (Fig 4A). These cells were transfected with a plasmid containing the full *BPIFA1* sequence in order to drive the expression of human BPIFA1. After controlling for protein load by quantifying total protein, subsequent immunoblotting confirmed that BPIFA1 was produced and secreted by these cells, and that this production was proportional to the amount of BPIFA1 plasmid transfected into the cells (Fig 4A). After allowing BPIFA1 to accumulate in the medium overnight, IB3-1 cells were stimulated with live *P*. *aeruginosa* for 4 hours to test whether the secreted BPIFA1 had antimicrobial properties when secreted in the context of airway epithelial cells. Over-expression of BPIFA1 using either 100 or 500 ng of transfected plasmid did not result in a change in CFU counts compared to transfection with empty vector (Fig 4B).

To test whether secreted BPIFA1 resulted in altered inflammatory responses, levels of IL-6 and IL-8, which are known to be dysregulated in CF [36], were measured. However, IL-6 and IL-8 levels were not significantly altered by BPIFA1 overexpression (Fig 4C).

### Effect of BPIFA1 and BPIFB1 on production of inflammatory cytokines by airway epithelial cells

**Fig 5:**
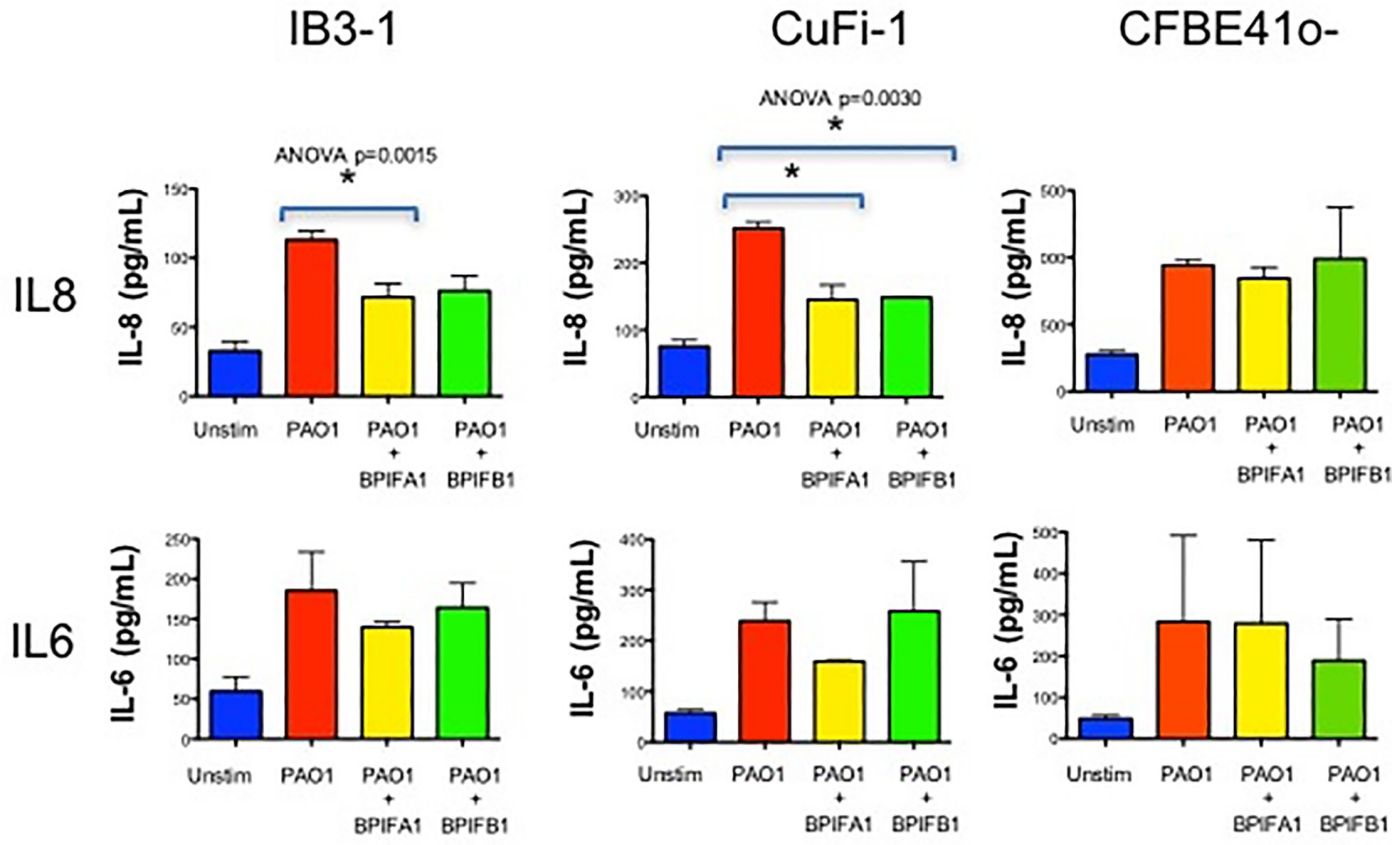
Quantification of inflammatory cytokine production by airway epithelial cells pretreated with recombinant BPIFA1 or BPIFB1 prior to bacterial stimulation. Quantification of IL-8 and IL-6 by ELISA produced by IB3-1, CuFi-1 and CFBE41o- airway epithelial cell lines that were unstimulated, stimulated with heat-killed *P*. *aeruginosa*, pretreated with recombinant BPIFA1 for 30 min followed by stimulation with heat-killed *P*. *aeruginosa*, or pretreated with recombinant BPIFB1 for 30 min followed by stimulation with heat-killed *P*. *aeruginosa*. ANOVA was used to identify statistical differences in IL-6 and IL-8 levels between conditions with * denoting *P* < 0.05.

BPIFA1/B1 have also been suggested to have anti-inflammatory capacity [16, 17]. To determine if BPIFA1 and BPIFB1 have an anti-inflammatory effect on airway epithelial cells during infection, three different CF airway epithelial cell lines were pretreated with recombinant BPIFA1 or BPIFB1 prior to stimulation with *P*. *aeruginosa*. Pre-treatment of IB3-1 and CuFi-1 with recombinant BPIFA1 prior to stimulation with PAO1 resulted in a reduction in IL-8 production (*P* = 0.002 and 0.003, respectively)(Fig 5). Furthermore, pretreatment with BPIFB1 was associated with decreased production of IL-8 in CuFi-1cells (*P* = 0.003). However, BPIFA1 and BPIFB1 pretreatment was not associated with reduction of IL-6 production in any cell type (Fig 5). We next sought to follow-up on this potential immunomodulatory activity using global transcriptomic analysis.

### Effect of BPIFA1 and BPIFB1 treatment on gene expression in CF airway epithelial cells

RNA-Seq was used to characterize the effect of BPIFA1 or BPIFB1 on the airway epithelial cell transcriptome. RNA samples from IB3-1 cells treated with recombinant BPIFA1 or BPIFB1 alone or followed by stimulation with *P*. *aeruginosa* were sequenced. Principal component analysis plots showing global changes in gene expression (Fig A in S1 File) demonstrated the global transcriptional impact of exposure to BPIFA1 and BPIFB1.

PAO1 stimulation resulted in the differential expression of 182 genes in IB3-1 cells, 122 that were upregulated and 60 downregulated (Table A in S1 File). Sigora was used to identify overrepresented pathways in the differentially expressed genes that responded to PAO1 stimulation. Pathway analysis identified the ‘JAK-STAT Signaling’ pathway (a pathway well known to control inflammation and immunoresponsiveness) to be overrepresented in the genes that are differentially expressed following exposure to *P*. *aeruginosa* (Table B in S1 File). This pathway includes genes such as Caspase 1 (*CASP1*), which is a proenzyme which can cleave and activate the inactive precursors of interleukin 1 and Interferon Lambda Receptor 1 (IFNLR1), which forms part of a receptor complex that interacts with several cytokines.

Treatment of IB3-1 cells with recombinant BPIFA1 alone or recombinant BPIFB1 alone resulted in large changes to the global transcriptome, with 1110 genes responding to BPIFA1 and 1324 genes responding to BPIFB1 compared to untreated cells (S1 Table). Pathway analysis identified several overrepresented pathways related to Rho GTPases (Table 3). The two genes with the greatest fold change in expression after BPIFA1 treatment were Keratin 17 (*KRT17*) and amyloid beta precursor like protein 1 (*APLP1*), while BPIFB1 treatment was associated with the greatest change in Pentraxin 3 (*PTX3*) and Sprouty related EVH1 domain containing 1 (*SRED1*).

**Table 3:** Overrepresented pathways by Sigora analysis in genes that were differentially expressed in response to BPIFA1 or BPIFB1 treatment compared to unstimulated cells.

Comparison	Pathway	*P* value
Unstimulated *vs*. BPIFA1	Signaling by Rho GTPases	1.38 × 10^−16^
	Rho GTPase cycle	6.93 × 10^−13^
	Infectious disease	2.66 × 10^−7^
	Rho GTPase Effectors	2.45 × 10^−6^
	SRP-dependent cotranslational targeting to membrane	8.16 × 10^−5^
	KSRP (KHSRP) binds and destabilizes mRNA	5.10 × 10^−4^
	Downstream signaling of activated FGFR1	0.019
	Cyclin A/B1 associated events during G2/M transition	0.050
Unstimulated *vs*. BPIFB1	Oxygen-dependent proline hydroxylation of hypoxia-inducible factor alpha	3.49 × 10^−9^
	Downregulation of TGF-beta receptor signaling	1.42 × 10^−8^
	Transcription of the HIV genome	4.07 × 10^−8^
	KSRP (KHSRP) binds and destabilizes mRNA	1.79 × 10^−6^
	Metabolism of vitamins and cofactors	3.00 × 10^−5^
	RNA Polymerase II Transcription	6.52 × 10^−5^
	Rho GTPase Effectors	1.87 × 10^−4^
	Metabolism of polyamines	9.76 × 10^−4^
	Constitutive signaling by NOTCH1 PEST domain mutants	1.79 × 10^−3^
	Cleavage of growing transcript in the termination region	3.10 × 10^−3^
	Signaling by Rho GTPases	3.76 × 10^−3^
	Golgi associated vesicle biogenesis	4.35 × 10^−6^
	Cyclin A/B1 associated events during G2/M transition	0.010
	Regulation of cholesterol biosynthesis by SREBP (SREBF)	0.035
	Chondroitin sulfate/dermatan sulfate metabolism	0.040
	Golgi cisternae pericentriolar stack reorganization	0.045

NetworkAnalyst [34] was used to analyze genes that were differentially expressed after treatment with BPIFA1 or BPIFB1 compared to baseline in order to create zero order (including interaction between differentially expressed genes only) protein:protein networks (Figs. B and C in S1 File). These networks included 373 seed proteins for BPIFA1 and 513 seed proteins for BPIFB1. Biological function enrichment analysis indicated that the BPIFB1 (Table C in S1 File) but not BPIFA1 (Table D in S1 File) network was enriched for three Reactome pathways related to TGF-beta signaling.

To corroborate the findings from IB3-1 cells, RNA was sequenced from an additional CF airway epithelial cell line, CFBE41o-, that was similarly treated with recombinant BPIFA1 and BPIFB1. A first order network (including interaction between seed proteins as well as proteins that are known to interact with them) was plotted due to fewer differentially expressed genes. This resulted in a network including 292 nodes (Fig D in S1 File) that was enriched for biological pathways involved in immunoresponsiveness, including ‘Influenza infection’ and ‘TRAF6 mediated NF-kB activation’ (Table E in S1 File). The BPIFB1 network from CFBE41o- cells was a first order network consisting of 255 nodes (Fig E in S1 File) and was enriched for immunological pathways including ‘Influenza Infection’, ‘TRAF6 mediated NF-kB activation’, ‘RIG-I/MDA5 mediated induction of IFN-alpha/beta pathways’ and ‘DAI mediated induction of type I IFNs’ (Table F in S1 File). These findings indicate that treatment of CF airway epithelial cells with BPIFA1 or BPIFB1 results in activation of the innate immune response.

To explore the mechanism by which BPIFA1 and BPIFB1 may modulate the immune response to *P*. *aeruginosa* infection we compared gene expression in cells pretreated with BPIFA1 or BPIFB1 prior to stimulation with *P*. *aeruginosa* to that of IB3-1 cells that were not pretreated. We found that pretreatment with BPIFA1 resulted in 66 differentially expressed genes compared to cells that were not pre-treated (Table G in S1 File) and pretreatment with BPIFB1 resulted in 279 differentially expressed genes compared to cells that were not pretreated (Table H in S1 File). Sigora analysis did not find any pathways to be overrepresented in genes that were differentially expressed in IB3-1 cells pretreated with BPIFA1 prior to *P*. *aeruginosa* stimulation compared to cells that were only stimulated with *P*. *aeruginosa*. In cells that were pretreated with BPIFB1 prior to stimulation, compared to cells that were stimulated with *P*. *aeruginosa* alone, Sigora identified ‘Gap junction trafficking and regulation’, ‘Platelet degranulation’, and ‘Metabolism of vitamins and cofactors pathways’ as overrepresented pathways (Table I in S1 File). A heatmap showing the genes that respond to PAO1 stimulation illustrates that BPIFA1 or BPIFB1 pretreatment did not affect their response to stimulation (Fig A in S1 File).

## Discussion

The discovery, validation, and functional characterization of modifier genes has shed light on disease pathophysiology in CF and is informing the development of novel therapies to benefit those living with the disease [37]. We have previously shown that a polymorphism in the *BPIFA1/BPIFB1* region was associated with lung disease severity in CF [4]. However, replication of genetic association findings is critical due to the high rate of false positives. A genetic association finding is not confirmed without replication in an independent cohort. In this study, we replicated the association of rs1078761 in an independent group of CF individuals. Furthermore, a meta-analysis of the discovery and replication cohorts demonstrated that the rs1078761 polymorphism remains the most significant association in the region with a *P* value of 2.39×10^−5^. This suggests that rs1078761 may be causative for the association.

We previously tested the effect of both rs1078761 and rs750064 on BPIFA1 gene and protein expression [4]. While rs750064 was found to have a lower p-value in association with BPIFA1 expression levels in a combined analysis of CF and non-CF controls, we found that in a stratified analysis rs1078761 had a greater effect size and lower p-value in CF subjects alone, while rs750064 had a greater effect size and lower p-value in non-CF controls [4]. This suggests that rs1078761 is more relevant as an eQTL in the unique situation of CF, and therefore rs750064 was not in the focus of this follow-up study.

To understand the mechanisms through which rs1078761 may associate with modification of CF lung disease, we quantified how genetic variation at this locus related to the expression of both BPIFA1 and BPIFB1 protein. Using saliva from clinically stable patients with CF, we found that CF patients who were homozygous for the G allele—the genotype associated with the most rapid decline in lung function—had significantly reduced levels of BPIFA1 protein, but not BPIFB1 levels. These data suggest that that BPIFA1 may be the causative gene within the locus for the association with CF lung disease severity. One potential weakness of this analysis is that we are detecting secreted BPIFA1 present in the saliva rather than the airways. BPIFA1 in saliva is likely to have been expressed by non-ciliated epithelial cells and mucous cells in the tonsil, tongue and parotid salivary glands [1, 38]. However, since the oral cavity is an entry way to the respiratory tract, salivary proteins can offer a first line of defense against airway infection. Therefore, in addition to the fact that salivary BPIFA1 levels may be representative of levels in airway secretions, the discovery that BPIFA1 protein levels in saliva vary in association with rs1078761 genotype is in itself important. Salivary samples offer an easily obtained and reproducible sample for quantification of BPIFA1 levels. Alternative methods of sampling airway secretions such as collection of sputum, bronchoalveolar lavage, or nasal washes are more invasive, can result in sample dilution or contamination, and can introduce bias by sampling different parts of the airways between subjects.

Previous studies have found that BPIFA1 has direct antimicrobial properties on a variety of bacteria [7–10, 13, 14, 35], although there is not unanimous support for this conclusion [13, 39]. Therefore, we investigated whether BPIFA1 and BPIFB1 could inhibit growth of the classic CF bacterial pathogen, *P*. *aeruginosa*. We found that recombinant BPIFA1 and BPIFB1 proteins do not inhibit growth *P*. *aeruginosa* (PAO1 strain), as measured by optical density or CFU counts. In addition, we found that recombinant BPIFA1 produced by CF airway epithelial cells also does not inhibit colony formation by *P*. *aeruginosa*. Together these data indicate that BPIFA1 and BPIFB1 do not have bacteriostatic or bactericidal activity against *P*. *aeruginosa*. A potential reason for the discrepancy in published studies relating to antimicrobial activity of BPIFA1 may be the use of recombinant BPIFA1 protein produced in *E*.*coli*. Since *E*.*coli* normally lack the capacity for protein glycosylation [40], and BPIFA1 is a highly glycosylated protein, the recombinant protein produced in eukaryotic organisms may be significantly different from the one produced in *E*.*coli*, both structurally and functionally [41]. Our experiments utilized BPIFA1 produced in yeast as well as overexpression assays using a BPIFA1 plasmid introduced into human airway epithelial cells.

Beyond antimicrobial activity, reducing lung-damaging inflammation is another mechanisms through which BPIFA1 and BPIFA2 may modify lung disease in CF [42]. Examining the immunomodulatory capacity of BPIFA1 and BPIFB1, we found that the addition of recombinant BPIFA1 protein to CF airway epithelial cells prior to stimulation with *P*. *aeruginosa* resulted in reduced production of IL-8 but not IL-6. Similarly, treatment with recombinant BPIFB1 protein prior to *P*. *aeruginosa* stimulation resulted in reduced production of IL-8 but not IL-6 in CuFi-1 cells. These results suggest that BPIFA1 and BPIFB1 may have anti-inflammatory properties in response to *P*. *aeruginosa* infection. These results are the first human validation of the anti-inflammatory actions of BPIFA1 or BPIFB1 that have been demonstrated in mouse models [13, 16, 17, 43].

To investigate the immunomodulatory mechanism of action of BPIFA1 and BPIFB1, we performed whole transcriptome sequencing of airway epithelial cells pretreated with recombinant BPIFA1 or BPIFB1 with or without stimulation with *P*. *aeruginosa*. Although the cells were responsive to stimulation with *P*. *aeruginosa*, we found that pretreatment with BPIFA1 and BPIFB1 had little effect on the response to infection. However, we did find that treatment of airway epithelial cells with BPIFA1 or BPIFB1 in the absence of stimulation had a large effect on the transcriptome. Pathway overrepresentation analysis revealed that both BPIFA1 and BPIFB1 activated several pathways related to Rho GTPases. Rho GTPases have been shown to play a central role in cellular migration [44], including in the recruitment of neutrophils [45]. This suggests that both BPIFA1 and BPIFB1 may play a role in chemotaxis of inflammatory cells, which is supported by data indicating that BPIFA1 may function in neutrophil recruitment [7]. ]

As an independent but complementary method of identifying biological functions that were enriched in genes differentially expressed in response to BPIFA1 and BPIFB1 treatment, the systems biology tool, NetworkAnalyst [34], was used to identify genes with known protein:protein interactions by generating a zero order network. Biological function enrichment analysis of the network indicated that the BPIFB1 network was enriched for Reactome pathways related to TGF-beta signaling, indicating that the genes differentially expressed in response to BPIFB1 were mainly involved in immune signaling. One limitation of the RNASeq analysis performed in our study is the use of a single sample in each condition which may limit the robustness of the data. Furthermore, since the cells were grown in a monolayer and lacked the cellular differentiation normally seen in the airway epithelium, these cell-lines may have responded differently than expected to BPIFA1 protein.

Taken together, our data favor BPIFA1 being responsible for the association with CF lung disease severity, since rs1078761 genotype was associated with variation in BPIFA1 protein levels in CF saliva. However, BPIFA1 and BPIFB1 were both able to reduce IL-8 production in response to *P*. *aeruginosa* infection and RNA-Seq data indicated that both molecules modulate the function of CF airway epithelia cells. It is possible that BPIFA1 is directly regulated by the rs1078761 polymorphism and is causative for the genetic association with CF severity, but that it functions synergistically with BPIFB1, so both molecules may play an important role in the disease.

## Conclusions

We have generated several lines of new evidence supporting a role for BPIFA1 and BPIFB1 in modulating the inflammatory response in CF, and have demonstrated that these molecules may contribute to CF severity through several complementary functions. Furthermore, our data support a new paradigm by which BPIFA1 and BPIFB1 may contribute to CF lung disease severity, resulting in gene expression changes in CF airway epithelial cells that could influence cell migration through Rho GTPase pathways and also by altering the response to viral infection.

## Supporting information

S1 File Supporting information file containing nine supplementary tables (A-I) and five supplementary figures (A-E).
(DOCX)

S1 Table Differentially expressed genes after BPIFA1 or BPIFB1 treatment of IB31 cells. (XLSX)
