# Supplementary material for: Immunomodulatory function of the cystic fibrosis modifier gene BPIFA1
Source: PLoS One. 2020 Jan 13;15(1):e0227067. doi: 10.1371/journal.pone.0227067 (PMC6957340; doi:10.1371/journal.pone.0227067)
Supplement: S1 File — Supporting information file containing nine supplementary tables (A-I) and five supplementary figures (A-E). (DOCX) [file pone.0227067.s001.docx]

**SUPPLEMENTARY DATA**

**Immunomodulatory function of the cystic fibrosis modifier gene *BPIFA1***

Aabida Saferali^1-4^, Anthony C. Tang^2^, Lisa J. Strug^5^, Bradley S. Quon^1^, James Zlosnik^2^, Andrew J. Sandford^1^, Stuart E. Turvey^2^

^1^Centre for Heart Lung Innovation, University of British Columbia and St Paul’s Hospital, Vancouver, British Columbia, Canada

^2^Department of Pediatrics, University of British Columbia and BC Children’s Hospital, Vancouver, British Columbia, Canada

^3^Channing Division of Network Medicine, Brigham and Women’s Hospital, Boston, Massachusetts, USA

^4^Harvard Medical School, Boston, Massachusetts, USA

^5^Program in Genetics and Genome Biology, The Hospital for Sick Children, Division of Biostatistics, Dalla Lana School of Public Health, University of Toronto, Toronto, Ontario, Canada

Table of Contents

[Table A 3](#_Toc28611489)

[Table B 6](#_Toc28611490)

[Table C 7](#_Toc28611491)

[Table D 8](#_Toc28611492)

[Table E 9](#_Toc28611493)

[Table F 12](#_Toc28611494)

[Table G 14](#_Toc28611495)

[Table H 16](#_Toc28611496)

[Table I 20](#_Toc28611497)

[Figure A 21](#_Toc28611498)

[Figure B 22](#_Toc28611499)

[Figure C 23](#_Toc28611500)

[Figure D 24](#_Toc28611501)

[Figure E 25](#_Toc28611502)

Table A**:** Genes that were differentially expressed in response to stimulation with PAO1 in IB3-1 cells.

| **Upregulated Gene** | **Log2 Fold Change** | ***P* value** | **Downregulated Gene** | **Log2 Fold Change** | ***P* value** |
| --- | --- | --- | --- | --- | --- |
| *C14orf132* | -0.827 | 0.008 | *HIST1H1C* | 0.586 | 0.066 |
| *LBH* | -0.813 | 0.011 | *MATN2* | 0.586 | 0.053 |
| *GPR1* | -0.809 | 0.009 | *PLK1* | 0.587 | 0.053 |
| *RGS2* | -0.796 | 0.012 | *TFB2M* | 0.587 | 0.066 |
| *IFI6* | -0.774 | 0.012 | *L3HYPDH* | 0.589 | 0.064 |
| *IFI44* | -0.759 | 0.017 | *ITGB8* | 0.590 | 0.066 |
| *SUSD1* | -0.759 | 0.015 | *MPP6* | 0.590 | 0.052 |
| *HPCAL4* | -0.742 | 0.011 | *CCNB1* | 0.590 | 0.059 |
| *COL4A6* | -0.731 | 0.018 | *CLK4* | 0.592 | 0.065 |
| *ZNF274* | -0.723 | 0.021 | *BACH1* | 0.596 | 0.062 |
| *HTR1D* | -0.723 | 0.019 | *PIK3CA* | 0.597 | 0.057 |
| *CDH6* | -0.720 | 0.021 | *FNBP1* | 0.597 | 0.042 |
| *MYO1D* | -0.719 | 0.025 | *HIST1H1D* | 0.597 | 0.032 |
| *MR1* | -0.703 | 0.027 | *ZNF26* | 0.600 | 0.058 |
| *RARRES3* | -0.702 | 0.019 | *IRS2* | 0.604 | 0.060 |
| *SBK1* | -0.698 | 0.027 | *ALG2* | 0.606 | 0.043 |
| *NSUN5P1* | -0.697 | 0.029 | *SLC25A32* | 0.606 | 0.059 |
| *HDHD3* | -0.695 | 0.030 | *ADAMTS16* | 0.607 | 0.045 |
| *VSIG10L* | -0.693 | 0.028 | *PDK1* | 0.607 | 0.055 |
| *ATOH8* | -0.693 | 0.028 | *NOG* | 0.608 | 0.035 |
| *LAMP3* | -0.692 | 0.030 | *ITPR1* | 0.609 | 0.048 |
| *CASP1* | -0.690 | 0.031 | *ELL2* | 0.610 | 0.054 |
| *P2RX4* | -0.689 | 0.032 | *SGOL2* | 0.615 | 0.054 |
| *ASS1* | -0.686 | 0.032 | *HIST1H2BD* | 0.616 | 0.019 |
| *SLPI* | -0.684 | 0.015 | *PRKCH* | 0.616 | 0.047 |
| *RAP1GAP2* | -0.682 | 0.029 | *PCF11* | 0.618 | 0.051 |
| *RPS6KA5* | -0.681 | 0.032 | *EGLN1* | 0.620 | 0.041 |
| *SLC1A3* | -0.681 | 0.034 | *FOXA1* | 0.625 | 0.038 |
| *C21orf58* | -0.680 | 0.031 | *ZBTB37* | 0.628 | 0.034 |
| *MFSD3* | -0.676 | 0.035 | *SMIM11* | 0.628 | 0.046 |
| *E2F1* | -0.676 | 0.030 | *PSPC1* | 0.634 | 0.044 |
| *MLLT11* | -0.674 | 0.035 | *RNF217* | 0.637 | 0.043 |
| *MDK* | -0.671 | 0.036 | *HIST1H3B* | 0.637 | 0.005 |
| *GCH1* | -0.671 | 0.034 | *TRIB2* | 0.639 | 0.044 |
| *H1F0* | -0.670 | 0.032 | *FAM20C* | 0.642 | 0.042 |
| *MXD3* | -0.669 | 0.037 | *LRIG2* | 0.644 | 0.042 |
| *ANO8* | -0.669 | 0.035 | *H19* | 0.648 | 0.033 |
| *APLP1* | -0.667 | 0.037 | *LIF* | 0.648 | 0.035 |
| *TMEM80* | -0.666 | 0.035 | *KIF18A* | 0.650 | 0.043 |
| *OASL* | -0.665 | 0.034 | *SCNN1A* | 0.650 | 0.036 |
| *MAP2* | -0.664 | 0.023 | *FBXO30* | 0.658 | 0.032 |
| *GALNT16* | -0.663 | 0.030 | *PDE1C* | 0.659 | 0.040 |
| *GRTP1* | -0.662 | 0.039 | *PPP1R10* | 0.662 | 0.030 |
| *EDN2* | -0.659 | 0.032 | *ZNF121* | 0.663 | 0.037 |
| *STARD4* | -0.658 | 0.040 | *HIST1H2BH* | 0.664 | 0.038 |
| *HLA-H* | -0.658 | 0.035 | *DTD2* | 0.669 | 0.037 |
| *SSTR1* | -0.656 | 0.041 | *HIST1H2AM* | 0.669 | 0.014 |
| *PKIA* | -0.655 | 0.024 | *ZNF469* | 0.675 | 0.032 |
| *CGN* | -0.653 | 0.042 | *NFATC2* | 0.686 | 0.028 |
| *WIPF1* | -0.653 | 0.030 | *SLC7A5* | 0.688 | 0.020 |
| *FBXO32* | -0.652 | 0.041 | *STK17A* | 0.689 | 0.032 |
| *CCNE2* | -0.650 | 0.042 | *SYT12* | 0.697 | 0.030 |
| *DERL3* | -0.649 | 0.035 | *NUP35* | 0.698 | 0.028 |
| *ANKRD44* | -0.647 | 0.035 | *CABLES1* | 0.725 | 0.022 |
| *SLC6A16* | -0.644 | 0.028 | *SPRY4* | 0.726 | 0.021 |
| *TMEM38A* | -0.639 | 0.038 | *TRIB1* | 0.748 | 0.020 |
| *GATSL3* | -0.638 | 0.037 | *JUN* | 0.756 | 0.019 |
| *TMEM59L* | -0.638 | 0.038 | *HIST1H1B* | 0.763 | 0.003 |
| *DAGLA* | -0.638 | 0.045 | *MYC* | 0.787 | 0.014 |
| *TMPO-AS1* | -0.636 | 0.047 | *TNFRSF10D* | 0.806 | 0.010 |
| *PAQR6* | -0.636 | 0.041 |  |  |  |
| *CCDC84* | -0.636 | 0.048 |  |  |  |
| *LOC148413* | -0.636 | 0.044 |  |  |  |
| *KRT17* | -0.634 | 0.048 |  |  |  |
| *NDRG2* | -0.634 | 0.033 |  |  |  |
| *GREB1L* | -0.633 | 0.049 |  |  |  |
| *LEPR* | -0.631 | 0.048 |  |  |  |
| *ARMCX2* | -0.631 | 0.048 |  |  |  |
| *TMEM198B* | -0.631 | 0.048 |  |  |  |
| *TMEM129* | -0.628 | 0.046 |  |  |  |
| *KLHL17* | -0.627 | 0.045 |  |  |  |
| *FAM57B* | -0.624 | 0.027 |  |  |  |
| *LOC100507002* | -0.623 | 0.043 |  |  |  |
| *RUSC1* | -0.621 | 0.033 |  |  |  |
| *FAM131A* | -0.621 | 0.048 |  |  |  |
| *DDX60* | -0.617 | 0.054 |  |  |  |
| *TSPAN15* | -0.616 | 0.052 |  |  |  |
| *FOXD2-AS1* | -0.615 | 0.055 |  |  |  |
| *DCHS1* | -0.615 | 0.042 |  |  |  |
| *KIAA1522* | -0.615 | 0.035 |  |  |  |
| *LTBP2* | -0.613 | 0.050 |  |  |  |
| *ZSCAN16-AS1* | -0.612 | 0.056 |  |  |  |
| *TMEM132A* | -0.612 | 0.037 |  |  |  |
| *ZNF251* | -0.612 | 0.051 |  |  |  |
| *ASB16-AS1* | -0.612 | 0.056 |  |  |  |
| *PTCHD4* | -0.611 | 0.047 |  |  |  |
| *NFKBID* | -0.610 | 0.058 |  |  |  |
| *PROS1* | -0.609 | 0.051 |  |  |  |
| *HSD11B1L* | -0.609 | 0.057 |  |  |  |
| *GAS1* | -0.608 | 0.036 |  |  |  |
| *BCORL1* | -0.608 | 0.036 |  |  |  |
| *RAB38* | -0.608 | 0.057 |  |  |  |
| *PLEKHN1* | -0.607 | 0.056 |  |  |  |
| *LOC284454* | -0.606 | 0.057 |  |  |  |
| *HLA-B* | -0.606 | 0.050 |  |  |  |
| *APCDD1* | -0.605 | 0.050 |  |  |  |
| *IRF9* | -0.605 | 0.060 |  |  |  |
| *ARMCX1* | -0.605 | 0.051 |  |  |  |
| *STAT2* | -0.605 | 0.052 |  |  |  |
| *FGD4* | -0.603 | 0.052 |  |  |  |
| *SLC22A17* | -0.603 | 0.058 |  |  |  |
| *LOC728392* | -0.602 | 0.059 |  |  |  |
| *RAB26* | -0.601 | 0.036 |  |  |  |
| *PARP14* | -0.600 | 0.062 |  |  |  |
| *OAS2* | -0.600 | 0.058 |  |  |  |
| *ZBED8* | -0.600 | 0.062 |  |  |  |
| *PWWP2B* | -0.599 | 0.057 |  |  |  |
| *NBR2* | -0.599 | 0.061 |  |  |  |
| *TRIM65* | -0.599 | 0.052 |  |  |  |
| *PID1* | -0.598 | 0.063 |  |  |  |
| *IFNLR1* | -0.597 | 0.059 |  |  |  |
| *SLC29A4* | -0.597 | 0.060 |  |  |  |
| *C8orf48* | -0.597 | 0.048 |  |  |  |
| *HLA-F* | -0.595 | 0.064 |  |  |  |
| *USP2* | -0.595 | 0.064 |  |  |  |
| *ARHGEF25* | -0.595 | 0.057 |  |  |  |
| *KREMEN2* | -0.594 | 0.064 |  |  |  |
| *ABAT* | -0.592 | 0.065 |  |  |  |
| *CTF1* | -0.590 | 0.054 |  |  |  |
| *OSR2* | -0.590 | 0.063 |  |  |  |
| *AUTS2* | -0.588 | 0.064 |  |  |  |
| *TMEM54* | -0.586 | 0.068 |  |  |  |

Table B: Pathways identified through Sigora analysis of genes differentially expressed in response to PAO1 stimulation of IB3-1 cells

| **Pathway** | ***P* value (Unadjusted)** | ***P* value (Adjusted)** | **Signature Genes** |
| --- | --- | --- | --- |
| Jak-STAT signaling pathway | 1.591 × 10^-4^ | 0.045 | *CASP1*  *CTF1*  *E2F1*  *HIST1H2BH*  *HLA-B*  *HTR1D*  *IFNLR1*  *IRF9*  *LEPR*  *LIF*  *MYC*  *NFATC2*  *P2RX4*  *PIK3CA*  *RPS6KA5*  *SSTR1*  *STAT2* |

Table C**:** Reactome pathways identified through biological function enrichment of protein-protein networks of differentially expressed genes in response to BPIFB1 treatment of IB31 cells

| **Pathway** | **Total** | **Expected** | **Hits** | ***P* value** | **FDR** |
| --- | --- | --- | --- | --- | --- |
| Cell Cycle, Mitotic | 411 | 12.6 | 31 | 2.11 × 10^-6^ | 0.00296 |
| Cell Cycle | 508 | 15.5 | 34 | 9.27 × 10^-6^ | 0.00553 |
| Downregulation of TGF-beta receptor signaling | 27 | 0.825 | 7 | 1.18 × 10^-5^ | 0.00553 |
| Mitotic M-M/G1 phases | 266 | 8.13 | 22 | 1.77 × 10^-5^ | 0.00586 |
| Mitotic G1-G1/S phases | 140 | 4.28 | 15 | 2.12 × 10^-5^ | 0.00586 |
| TGF-beta receptor signaling activates SMADs | 30 | 0.917 | 7 | 2.51 × 10^-5^ | 0.00586 |
| Signaling by TGF-beta Receptor Complex | 70 | 2.14 | 9 | 0.000251 | 0.0421 |
| M Phase | 233 | 7.12 | 18 | 0.000252 | 0.0421 |

Table D**:** Reactome pathways identified through biological function enrichment of protein-protein networks of differentially expressed genes in response to BPIFA1 treatment of IB31 cells

| **Pathway** | **Total** | **Expected** | **Hits** | ***P* value** | **FDR** |
| --- | --- | --- | --- | --- | --- |
| Cell Cycle, Mitotic | 411 | 10.3 | 31 | 2.03 × 10^-8^ | 2.85 × 10^-5^ |
| Cell Cycle | 508 | 12.7 | 34 | 7.50 × 10^-8^ | 5.25 × 10^-5^ |
| Mitotic M-M/G1 phases | 266 | 6.65 | 20 | 9.10 × 10^-6^ | 0.00425 |
| Mitotic Prometaphase | 127 | 3.18 | 13 | 1.50 × 10^-5^ | 0.00527 |
| Resolution of Sister Chromatid Cohesion | 118 | 2.95 | 12 | 3.46 × 10^-5^ | 0.00969 |
| Mitotic G1-G1/S phases | 140 | 3.5 | 13 | 4.28 × 10^-5^ | 0.01 |
| G1/S Transition | 113 | 2.83 | 11 | 0.00011 | 0.0221 |
| Adaptive Immune System | 654 | 16.4 | 32 | 0.000141 | 0.0247 |
| Separation of Sister Chromatids | 186 | 4.65 | 14 | 0.000214 | 0.0307 |
| M Phase | 233 | 5.83 | 16 | 0.000219 | 0.0307 |
| Cyclin A/B1 associated events during G2/M transition | 14 | 0.35 | 4 | 0.000311 | 0.0396 |
| Mitotic Anaphase | 198 | 4.95 | 14 | 0.000408 | 0.0463 |
| Mitotic Metaphase and Anaphase | 199 | 4.98 | 14 | 0.000429 | 0.0463 |

Table E**:** Reactome pathways identified through biological function enrichment of protein-protein networks of differentially expressed genes in response to BPIFA1 treatment of CFBE41o- cells

| **Pathway** | **Total** | **Expected** | **Hits** | ***P* value** | **FDR** |
| --- | --- | --- | --- | --- | --- |
| Peptide chain elongation | 178 | 5.17 | 51 | 3.18 × 10^-38^ | 4.45 × 10^-35^ |
| Nonsense Mediated Decay Enhanced by the Exon Junction Complex | 203 | 5.9 | 53 | 1.85 × 10^-37^ | 8.46 × 10^-35^ |
| Nonsense-Mediated Decay | 203 | 5.9 | 53 | 1.85 × 10^-37^ | 8.46 × 10^-35^ |
| Influenza Viral RNA Transcription and Replication | 176 | 5.11 | 50 | 3.02 × 10^-37^ | 8.46 × 10^-35^ |
| Viral mRNA Translation | 176 | 5.11 | 50 | 3.02 × 10^-37^ | 8.46 × 10^-35^ |
| Eukaryotic Translation Elongation | 186 | 5.4 | 51 | 3.67 × 10^-37^ | 8.58 × 10^-35^ |
| Eukaryotic Translation Termination | 178 | 5.17 | 50 | 5.60 × 10^-37^ | 1.12 × 10^-34^ |
| Influenza Life Cycle | 180 | 5.23 | 50 | 1.03 × 10^-36^ | 1.80 × 10^-34^ |
| Nonsense Mediated Decay Independent of the Exon Junction Complex | 184 | 5.35 | 50 | 3.39 × 10^-36^ | 5.28 × 10^-34^ |
| Influenza Infection | 185 | 5.38 | 50 | 4.55 × 10^-36^ | 6.38 × 10^-34^ |
| Formation of a pool of free 40S subunits | 189 | 5.49 | 50 | 1.44 × 10^-35^ | 1.84 × 10^-33^ |
| GTP hydrolysis and joining of the 60S ribosomal subunit | 201 | 5.84 | 51 | 2.58 × 10^-35^ | 2.58 × 10^-33^ |
| 3' -UTR-mediated translational regulation | 201 | 5.84 | 51 | 2.58 × 10^-35^ | 2.58 × 10^-33^ |
| L13a-mediated translational silencing of Ceruloplasmin expression | 201 | 5.84 | 51 | 2.58 × 10^-35^ | 2.58 × 10^-33^ |
| Eukaryotic Translation Initiation | 209 | 6.07 | 51 | 2.12 × 10^-34^ | 1.86 × 10^-32^ |
| Cap-dependent Translation Initiation | 209 | 6.07 | 51 | 2.12 × 10^-34^ | 1.86 × 10^-32^ |
| SRP-dependent cotranslational protein targeting to membrane | 204 | 5.93 | 50 | 8.39 × 10^-34^ | 6.92 × 10^-32^ |
| Translation | 249 | 7.23 | 52 | 1.83 × 10^-31^ | 1.42 × 10^-29^ |
| Metabolism of RNA | 339 | 9.85 | 59 | 2.82 × 10^-31^ | 2.08 × 10^-29^ |
| Metabolism of mRNA | 317 | 9.21 | 57 | 6.10 × 10^-31^ | 4.28 × 10^-29^ |
| Respiratory electron transport | 82 | 2.38 | 32 | 8.55 × 10^-29^ | 5.71 × 10^-27^ |
| Respiratory electron transport, ATP synthesis by chemiosmotic coupling, and heat production by uncoupling proteins. | 101 | 2.93 | 32 | 1.91 × 10^-25^ | 1.22 × 10^-23^ |
| The citric acid (TCA) cycle and respiratory electron transport | 145 | 4.21 | 32 | 4.31 × 10^-20^ | 2.63 × 10^-18^ |
| Formation of the ternary complex, and subsequently, the 43S complex | 83 | 2.41 | 23 | 4.93 × 10^-17^ | 2.88 × 10^-15^ |
| Ribosomal scanning and start codon recognition | 91 | 2.64 | 23 | 4.64 × 10^-16^ | 2.60 × 10^-14^ |
| Translation initiation complex formation | 92 | 2.67 | 23 | 6.03 × 10^-16^ | 3.25 × 10^-14^ |
| Activation of the mRNA upon binding of the cap-binding complex and eIFs, and subsequent binding to 43S | 93 | 2.7 | 23 | 7.81 × 10^-16^ | 4.05 × 10^-14^ |
| Gene Expression | 1090 | 31.6 | 76 | 6.32 × 10^-15^ | 3.17 × 10^-13^ |
| Metabolism of proteins | 689 | 20 | 58 | 1.43 × 10^-14^ | 6.94 × 10^-13^ |
| Disease | 945 | 27.5 | 66 | 1.06 × 10^-12^ | 4.97 × 10^-11^ |
| Assembly of the RAD51-ssDNA nucleoprotein complex | 5 | 0.145 | 5 | 1.97 × 10^-08^ | 8.90 × 10^-07^ |
| Homologous DNA pairing and strand exchange | 6 | 0.174 | 5 | 1.15 × 10^-07^ | 4.90 × 10^-06^ |
| Presynaptic phase of homologous DNA pairing and strand exchange | 6 | 0.174 | 5 | 1.15 × 10^-07^ | 4.90 × 10^-06^ |
| Removal of the Flap Intermediate | 14 | 0.407 | 6 | 1.38 × 10^-06^ | 5.68 × 10^-05^ |
| Processive synthesis on the lagging strand | 15 | 0.436 | 6 | 2.24 × 10^-06^ | 8.98 × 10^-05^ |
| Homologous recombination repair of replication-independent double-strand breaks | 16 | 0.465 | 6 | 3.50 × 10^-06^ | 0.000133 |
| Homologous Recombination Repair | 16 | 0.465 | 6 | 3.50 × 10^-06^ | 0.000133 |
| Removal of the Flap Intermediate from the C-strand | 10 | 0.291 | 5 | 4.40 × 10^-06^ | 0.000162 |
| Processive synthesis on the C-strand of the telomere | 11 | 0.32 | 5 | 7.88 × 10^-06^ | 0.000283 |
| Chromosome Maintenance | 124 | 3.6 | 14 | 1.25 × 10^-05^ | 0.00044 |
| Lagging Strand Synthesis | 20 | 0.581 | 6 | 1.54 × 10^-05^ | 0.000525 |
| Nectin/Necl trans heterodimerization | 7 | 0.203 | 4 | 2.26 × 10^-05^ | 0.000727 |
| DNA strand elongation | 31 | 0.901 | 7 | 2.28 × 10^-05^ | 0.000727 |
| Adherens junctions interactions | 31 | 0.901 | 7 | 2.28 × 10^-05^ | 0.000727 |
| Processing of DNA double-strand break ends | 3 | 0.0872 | 3 | 2.42 × 10^-05^ | 0.000753 |
| Telomere C-strand (Lagging Strand) Synthesis | 22 | 0.639 | 6 | 2.82 × 10^-05^ | 0.00085 |
| Activation of the pre-replicative complex | 32 | 0.93 | 7 | 2.85 × 10^-05^ | 0.00085 |
| Repair synthesis of patch ~27-30 bases long by DNA polymerase | 15 | 0.436 | 5 | 4.66 × 10^-05^ | 0.00133 |
| Repair synthesis for gap-filling by DNA polymerase in TC-NER | 15 | 0.436 | 5 | 4.66 × 10^-05^ | 0.00133 |
| Double-Strand Break Repair | 24 | 0.697 | 6 | 4.84 × 10^-05^ | 0.00133 |
| Extension of Telomeres | 24 | 0.697 | 6 | 4.84 × 10^-05^ | 0.00133 |
| Gap-filling DNA repair synthesis and ligation in GG-NER | 16 | 0.465 | 5 | 6.62 × 10^-05^ | 0.00175 |
| Gap-filling DNA repair synthesis and ligation in TC-NER | 16 | 0.465 | 5 | 6.62 × 10^-05^ | 0.00175 |
| Cell junction organization | 89 | 2.59 | 10 | 0.000235 | 0.0061 |
| Mitotic M-M/G1 phases | 266 | 7.73 | 19 | 0.000243 | 0.00619 |
| Cell-cell junction organization | 60 | 1.74 | 8 | 0.000308 | 0.00771 |
| G1/S Transition | 113 | 3.28 | 11 | 0.00041 | 0.0101 |
| Resolution of Sister Chromatid Cohesion | 118 | 3.43 | 11 | 0.000595 | 0.0144 |
| Cell Cycle | 508 | 14.8 | 28 | 0.000689 | 0.0164 |
| DNA Replication | 102 | 2.96 | 10 | 0.000709 | 0.0166 |
| Mitotic G1-G1/S phases | 140 | 4.07 | 12 | 0.000724 | 0.0166 |
| Cell Cycle, Mitotic | 411 | 11.9 | 24 | 0.000767 | 0.0173 |
| S Phase | 122 | 3.54 | 11 | 0.000789 | 0.0176 |
| TRAF6 mediated NF-kB activation | 16 | 0.465 | 4 | 0.000956 | 0.0209 |
| Nucleotide-binding domain, leucine rich repeat containing receptor (NLR) signaling pathways | 55 | 1.6 | 7 | 0.000976 | 0.0211 |
| Mitotic Prometaphase | 127 | 3.69 | 11 | 0.0011 | 0.0234 |
| Synthesis of DNA | 95 | 2.76 | 9 | 0.00167 | 0.0349 |
| DNA Replication Pre-Initiation | 80 | 2.32 | 8 | 0.00213 | 0.0432 |
| M/G1 Transition | 80 | 2.32 | 8 | 0.00213 | 0.0432 |
| Transcription-coupled NER (TC-NER) | 47 | 1.37 | 6 | 0.00222 | 0.0444 |

Table F**:** Reactome pathways identified through biological function enrichment of protein-protein networks of differentially expressed genes in response to BPIFB1 treatment of CFBE41o- cells

| **Pathway** | **Total** | **Expected** | **Hits** | ***P* value** | **FDR** |
| --- | --- | --- | --- | --- | --- |
| Formation of a pool of free 40S subunits | 189 | 4.67 | 70 | 1.97 × 10^-68^ | 2.77 × 10^-65^ |
| GTP hydrolysis and joining of the 60S ribosomal subunit | 201 | 4.97 | 70 | 3.39 × 10^-66^ | 1.19 × 10^-63^ |
| 3' -UTR-mediated translational regulation | 201 | 4.97 | 70 | 3.39 × 10^-66^ | 1.19 × 10^-63^ |
| L13a-mediated translational silencing of Ceruloplasmin expression | 201 | 4.97 | 70 | 3.39 × 10^-66^ | 1.19 × 10^-63^ |
| Eukaryotic Translation Initiation | 209 | 5.16 | 70 | 8.46 × 10^-65^ | 1.98 × 10^-62^ |
| Cap-dependent Translation Initiation | 209 | 5.16 | 70 | 8.46 × 10^-65^ | 1.98 × 10^-62^ |
| Influenza Viral RNA Transcription and Replication | 176 | 4.35 | 66 | 1.34 × 10^-64^ | 2.34 × 10^-62^ |
| Viral mRNA Translation | 176 | 4.35 | 66 | 1.34 × 10^-64^ | 2.34 × 10^-62^ |
| Influenza Infection | 185 | 4.57 | 67 | 1.90 × 10^-64^ | 2.96 × 10^-62^ |
| Eukaryotic Translation Elongation | 186 | 4.6 | 67 | 2.93 × 10^-64^ | 3.84 × 10^-62^ |
| Eukaryotic Translation Termination | 178 | 4.4 | 66 | 3.29 × 10^-64^ | 3.84 × 10^-62^ |
| Peptide chain elongation | 178 | 4.4 | 66 | 3.29 × 10^-64^ | 3.84 × 10^-62^ |
| Influenza Life Cycle | 180 | 4.45 | 66 | 7.97 × 10^-64^ | 8.60 × 10^-62^ |
| Nonsense Mediated Decay Independent of the Exon Junction Complex | 184 | 4.55 | 66 | 4.52 × 10^-63^ | 4.53 × 10^-61^ |
| Translation | 249 | 6.15 | 71 | 3.94 × 10^-60^ | 3.68 × 10^-58^ |
| Nonsense Mediated Decay Enhanced by the Exon Junction Complex | 203 | 5.02 | 66 | 9.24 × 10^-60^ | 7.62 × 10^-58^ |
| Nonsense-Mediated Decay | 203 | 5.02 | 66 | 9.24 × 10^-60^ | 7.62 × 10^-58^ |
| SRP-dependent cotranslational protein targeting to membrane | 204 | 5.04 | 66 | 1.35 × 10^-59^ | 1.05 × 10^-57^ |
| Metabolism of mRNA | 317 | 7.83 | 69 | 1.90 × 10^-49^ | 1.40 × 10^-47^ |
| Metabolism of RNA | 339 | 8.38 | 70 | 1.45 × 10^-48^ | 1.02 × 10^-46^ |
| Gene Expression | 1090 | 26.8 | 103 | 6.88 × 10^-42^ | 4.60 × 10^-40^ |
| Formation of the ternary complex, and subsequently, the 43S complex | 83 | 2.05 | 34 | 5.45 × 10^-34^ | 3.47 × 10^-32^ |
| Metabolism of proteins | 689 | 17 | 77 | 8.66 × 10^-34^ | 5.28 × 10^-32^ |
| Ribosomal scanning and start codon recognition | 91 | 2.25 | 34 | 2.40 × 10^-32^ | 1.40 × 10^-30^ |
| Translation initiation complex formation | 92 | 2.27 | 34 | 3.73 × 10^-32^ | 2.09 × 10^-30^ |
| Activation of the mRNA upon binding of the cap-binding complex and eIFs, and subsequent binding to 43S | 93 | 2.3 | 34 | 5.76 × 10^-32^ | 3.11 × 10^-30^ |
| Disease | 945 | 23.4 | 84 | 6.47 × 10^-30^ | 3.36 × 10^-28^ |
| RNA Polymerase III Chain Elongation | 18 | 0.445 | 11 | 4.12 × 10^-14^ | 1.99 × 10^-12^ |
| RNA Polymerase III Transcription Termination | 18 | 0.445 | 11 | 4.12 × 10^-14^ | 1.99 × 10^-12^ |
| RNA Polymerase III Transcription Initiation From Type 1 Promoter | 25 | 0.618 | 11 | 4.97 × 10^-12^ | 2.25 × 10^-10^ |
| RNA Polymerase III Transcription Initiation From Type 2 Promoter | 25 | 0.618 | 11 | 4.97 × 10^-12^ | 2.25 × 10^-10^ |
| RNA Polymerase III Transcription Initiation From Type 3 Promoter | 27 | 0.667 | 11 | 1.39 × 10^-11^ | 6.10 × 10^-10^ |
| RNA Polymerase III Transcription | 33 | 0.815 | 11 | 1.82 × 10^-10^ | 7.29 × 10^-09^ |
| RNA Polymerase III Transcription Initiation | 33 | 0.815 | 11 | 1.82 × 10^-10^ | 7.29 × 10^-09^ |
| RNA Polymerase III Abortive And Retractive Initiation | 33 | 0.815 | 11 | 1.82 × 10^-10^ | 7.29 × 10^-09^ |
| RNA Polymerase I, RNA Polymerase III, and Mitochondrial Transcription | 59 | 1.46 | 11 | 1.51 × 10^-07^ | 5.87 × 10^-06^ |
| Transcription | 149 | 3.68 | 14 | 1.64 × 10^-05^ | 0.000621 |
| TRAF6 mediated NF-kB activation | 16 | 0.395 | 5 | 3.04 × 10^-05^ | 0.00112 |
| Signaling by constitutively active EGFR | 19 | 0.47 | 5 | 7.62 × 10^-05^ | 0.00274 |
| RIP-mediated NFkB activation via DAI | 11 | 0.272 | 4 | 0.000104 | 0.00363 |
| mRNA Splicing | 115 | 2.84 | 11 | 0.000116 | 0.00387 |
| mRNA Splicing - Major Pathway | 115 | 2.84 | 11 | 0.000116 | 0.00387 |
| Signaling by Interleukins | 116 | 2.87 | 11 | 0.000125 | 0.00409 |
| Processing of Capped Intron-Containing Pre-mRNA | 119 | 2.94 | 11 | 0.000158 | 0.00503 |
| TAK1 activates NFkB by phosphorylation and activation of IKKs complex | 22 | 0.544 | 5 | 0.000162 | 0.00506 |
| DAI mediated induction of type I IFNs | 13 | 0.321 | 4 | 0.000216 | 0.00658 |
| RIG-I/MDA5 mediated induction of IFN-alpha/beta pathways | 67 | 1.66 | 8 | 0.000221 | 0.0066 |
| mRNA Processing | 140 | 3.46 | 11 | 0.000649 | 0.019 |
| Interleukin-1 signaling | 45 | 1.11 | 6 | 0.000762 | 0.0218 |
| Cytosolic sensors of pathogen-associated DNA | 19 | 0.47 | 4 | 0.00104 | 0.0292 |

Table G**:** Genes that are differentially expressed in response to BPIFA1 pretreatment prior to stimulation with PAO1 in IB3-1 cells

| **Upregulated Gene** | **Log2 Fold Change** | ***P* value** | **Downregulated Gene** | **Log2 Fold Change** | ***P* value** |
| --- | --- | --- | --- | --- | --- |
| *RPS27L* | 0.740 | 0.018 | *MIR6723* | -0.812 | 0.011 |
| *GBP1* | 0.712 | 0.026 | *ATF3* | -0.795 | 0.013 |
| *LINC00998* | 0.697 | 0.030 | *PABPN1* | -0.785 | 0.015 |
| *NR6A1* | 0.693 | 0.024 | *STC2* | -0.768 | 0.013 |
| *MLLT11* | 0.653 | 0.041 | *SLC3A2* | -0.746 | 0.017 |
| *LYSMD2* | 0.653 | 0.041 | *PPP1R15A* | -0.737 | 0.022 |
| *FLJ44635* | 0.642 | 0.042 | *DNLZ* | -0.734 | 0.022 |
| *ZFP36* | 0.640 | 0.044 | *GPT2* | -0.702 | 0.028 |
| *NMRK1* | 0.634 | 0.048 | *ZNF564* | -0.697 | 0.030 |
| *ULBP2* | 0.633 | 0.046 | *GADD45A* | -0.679 | 0.026 |
| *MSMO1* | 0.617 | 0.051 | *ERN1* | -0.677 | 0.033 |
| *UBL3* | 0.612 | 0.056 | *LINC00263* | -0.677 | 0.027 |
| *NTAN1* | 0.607 | 0.040 | *COBL* | -0.672 | 0.033 |
| *RGS2* | 0.604 | 0.057 | *PSAT1* | -0.668 | 0.036 |
| *TRIM58* | 0.600 | 0.062 | *SCAMP1-AS1* | -0.666 | 0.038 |
| *WDR83OS* | 0.592 | 0.060 | *CARS* | -0.661 | 0.039 |
|  |  |  | *ZNF559* | -0.654 | 0.040 |
|  |  |  | *VEGFA* | -0.649 | 0.027 |
|  |  |  | *GDF15* | -0.649 | 0.043 |
|  |  |  | *ZNF44* | -0.648 | 0.042 |
|  |  |  | *AGAP6* | -0.648 | 0.043 |
|  |  |  | *RGS3* | -0.644 | 0.045 |
|  |  |  | *ENDOG* | -0.637 | 0.047 |
|  |  |  | *CEBPG* | -0.636 | 0.047 |
|  |  |  | *AARS* | -0.631 | 0.045 |
|  |  |  | *LENG1* | -0.627 | 0.050 |
|  |  |  | *SLMO1* | -0.626 | 0.051 |
|  |  |  | *KDM4D* | -0.626 | 0.051 |
|  |  |  | *HSD3B7* | -0.623 | 0.051 |
|  |  |  | *WDR45* | -0.621 | 0.041 |
|  |  |  | *SNHG17* | -0.614 | 0.041 |
|  |  |  | *ARHGEF2* | -0.612 | 0.045 |
|  |  |  | *PINX1* | -0.612 | 0.041 |
|  |  |  | *MOCOS* | -0.611 | 0.057 |
|  |  |  | *LINC00963* | -0.608 | 0.058 |
|  |  |  | *SNHG7* | -0.607 | 0.056 |
|  |  |  | *C9orf169* | -0.607 | 0.044 |
|  |  |  | *ZNF530* | -0.606 | 0.059 |
|  |  |  | *NGF* | -0.604 | 0.046 |
|  |  |  | *ETS2* | -0.602 | 0.044 |
|  |  |  | *HARBI1* | -0.599 | 0.062 |
|  |  |  | *DDIT4* | -0.598 | 0.038 |
|  |  |  | *SMA5* | -0.597 | 0.019 |
|  |  |  | *TMEM186* | -0.596 | 0.052 |
|  |  |  | *ZNF256* | -0.595 | 0.038 |
|  |  |  | *SYCE2* | -0.594 | 0.063 |
|  |  |  | *C1RL-AS1* | -0.592 | 0.062 |
|  |  |  | *TIGD2* | -0.590 | 0.066 |
|  |  |  | *SNRNP35* | -0.588 | 0.059 |
|  |  |  | *SLC7A5* | -0.586 | 0.047 |

Table H**:** Genes that are differentially expressed in response to BPIFB1 pretreatment prior to stimulation with PAO1 in IB3-1 cells

| **Upregulated Gene** | **Log2 Fold Change** | ***P* value** | **Downregulated Gene** | **Log2 Fold Change** | ***P* value** |
| --- | --- | --- | --- | --- | --- |
| *MYL6* | 0.857 | 0.007 | *PSAT1* | -0.891 | 0.005 |
| *APLN* | 0.790 | 0.014 | *CBS* | -0.866 | 0.007 |
| *EFNB3* | 0.788 | 0.014 | *ALDH1L2* | -0.863 | 0.005 |
| *PIANP* | 0.770 | 0.013 | *MOCOS* | -0.816 | 0.011 |
| *ANP32C* | 0.769 | 0.016 | *SLC16A9* | -0.799 | 0.012 |
| *FAM196B* | 0.769 | 0.017 | *KIF21B* | -0.787 | 0.013 |
| *MT1X* | 0.762 | 0.015 | *CARS* | -0.786 | 0.014 |
| *HSPA1B* | 0.758 | 0.018 | *DDR2* | -0.777 | 0.004 |
| *RPS14P3* | 0.746 | 0.020 | *CEBPG* | -0.774 | 0.016 |
| *ESRRA* | 0.740 | 0.016 | *LOC101927045* | -0.772 | 0.009 |
| *HSPA8* | 0.736 | 0.021 | *GPT2* | -0.772 | 0.015 |
| *ATP5G1* | 0.733 | 0.017 | *ZCCHC8* | -0.770 | 0.014 |
| *SENP3* | 0.731 | 0.023 | *MORN1* | -0.763 | 0.016 |
| *PTGES* | 0.726 | 0.019 | *ENTPD5* | -0.758 | 0.018 |
| *TUBA4A* | 0.726 | 0.020 | *PCLO* | -0.750 | 0.019 |
| *ACTB* | 0.724 | 0.024 | *C1RL-AS1* | -0.744 | 0.019 |
| *HSPA1A* | 0.723 | 0.024 | *ERN1* | -0.741 | 0.020 |
| *UBE2E4P* | 0.716 | 0.026 | *SLFN5* | -0.740 | 0.020 |
| *PDE9A* | 0.711 | 0.027 | *SLC3A2* | -0.735 | 0.018 |
| *FLJ44635* | 0.710 | 0.024 | *AARS* | -0.734 | 0.020 |
| *HOXC13* | 0.707 | 0.027 | *SLC25A21-AS1* | -0.728 | 0.009 |
| *HHIP* | 0.703 | 0.029 | *LOC100132077* | -0.728 | 0.020 |
| *TAGLN2* | 0.701 | 0.018 | *ZNF26* | -0.725 | 0.022 |
| *PPP1R13L* | 0.691 | 0.025 | *ZNF558* | -0.724 | 0.022 |
| *FAM127A* | 0.691 | 0.021 | *YARS* | -0.723 | 0.013 |
| *NT5DC2* | 0.688 | 0.029 | *DHRS2* | -0.721 | 0.019 |
| *ARHGAP22* | 0.686 | 0.029 | *UBA6-AS1* | -0.720 | 0.025 |
| *MGLL* | 0.686 | 0.026 | *SMAD1* | -0.719 | 0.024 |
| *PTGER2* | 0.686 | 0.032 | *FLJ46906* | -0.718 | 0.023 |
| *EBPL* | 0.685 | 0.027 | *ELAC1* | -0.718 | 0.025 |
| *NPM3* | 0.679 | 0.031 | *MARS* | -0.718 | 0.020 |
| *FASN* | 0.679 | 0.024 | *FAM122C* | -0.716 | 0.023 |
| *NDUFA3* | 0.678 | 0.033 | *ZNF564* | -0.714 | 0.026 |
| *F2R* | 0.678 | 0.029 | *PGGT1B* | -0.714 | 0.025 |
| *SERPINE1* | 0.675 | 0.033 | *SERPINA3* | -0.711 | 0.019 |
| *CLCF1* | 0.674 | 0.035 | *OSCP1* | -0.709 | 0.027 |
| *ARL2* | 0.674 | 0.026 | *TUFT1* | -0.708 | 0.021 |
| *NT5E* | 0.672 | 0.024 | *TUBE1* | -0.707 | 0.028 |
| *MGC12916* | 0.669 | 0.016 | *ATF3* | -0.707 | 0.028 |
| *EIF5AL1* | 0.668 | 0.037 | *SLMO1* | -0.706 | 0.028 |
| *S100A16* | 0.668 | 0.029 | *FGFR2* | -0.703 | 0.027 |
| *NPIPB5* | 0.667 | 0.035 | *MTHFD2* | -0.702 | 0.025 |
| *RIMKLA* | 0.665 | 0.038 | *STC2* | -0.701 | 0.023 |
| *INSIG1* | 0.663 | 0.024 | *LINC00263* | -0.697 | 0.022 |
| *SLC29A1* | 0.661 | 0.023 | *ZNF518A* | -0.695 | 0.027 |
| *PFKM* | 0.660 | 0.022 | *SNHG12* | -0.693 | 0.030 |
| *PDCD1LG2* | 0.659 | 0.037 | *KLF4* | -0.691 | 0.030 |
| *PTTG3P* | 0.658 | 0.039 | *GDF15* | -0.690 | 0.031 |
| *LDLR* | 0.658 | 0.030 | *TADA2A* | -0.689 | 0.029 |
| *SNAI2* | 0.658 | 0.040 | *DZIP3* | -0.689 | 0.028 |
| *NF2* | 0.654 | 0.030 | *SLC6A9* | -0.688 | 0.019 |
| *GDF6* | 0.654 | 0.028 | *PPM1E* | -0.688 | 0.032 |
| *SEMA7A* | 0.653 | 0.038 | *METTL14* | -0.683 | 0.029 |
| *VEGFC* | 0.652 | 0.040 | *ZNF425* | -0.683 | 0.030 |
| *PLXNA2* | 0.651 | 0.040 | *CTH* | -0.682 | 0.030 |
| *NAV3* | 0.651 | 0.043 | *NBPF1* | -0.680 | 0.034 |
| *MIR31HG* | 0.650 | 0.043 | *SH3TC1* | -0.676 | 0.033 |
| *IMPDH1* | 0.647 | 0.028 | *LOC100132352* | -0.675 | 0.034 |
| *CRYZ* | 0.646 | 0.041 | *LMO4* | -0.674 | 0.036 |
| *LAMTOR4* | 0.646 | 0.033 | *CLUHP3* | -0.673 | 0.030 |
| *COL1A1* | 0.631 | 0.048 | *ZC3H6* | -0.667 | 0.038 |
| *CHPF2* | 0.630 | 0.046 | *TPK1* | -0.665 | 0.023 |
| *PODXL* | 0.630 | 0.031 | *ZNF528* | -0.663 | 0.029 |
| *SEMA3A* | 0.630 | 0.047 | *SOGA3* | -0.663 | 0.037 |
| *RAB3A* | 0.625 | 0.051 | *VEGFA* | -0.659 | 0.025 |
| *SLC6A6* | 0.624 | 0.041 | *SIRT5* | -0.658 | 0.036 |
| *ARHGDIB* | 0.624 | 0.041 | *ZFP41* | -0.657 | 0.038 |
| *WNT7B* | 0.623 | 0.052 | *KAZALD1* | -0.656 | 0.040 |
| *MIR100HG* | 0.621 | 0.029 | *ZSCAN31* | -0.656 | 0.031 |
| *TIMP1* | 0.621 | 0.034 | *SPEF2* | -0.652 | 0.042 |
| *VAT1L* | 0.618 | 0.053 | *UHRF1BP1* | -0.652 | 0.039 |
| *RPL27* | 0.617 | 0.043 | *NDUFA6-AS1* | -0.648 | 0.026 |
| *HM13* | 0.615 | 0.036 | *LINC00467* | -0.647 | 0.042 |
| *IDI1* | 0.614 | 0.037 | *ZNF460* | -0.645 | 0.033 |
| *GSTO1* | 0.613 | 0.039 | *CRCP* | -0.644 | 0.030 |
| *NOTCH3* | 0.613 | 0.055 | *SCAMP1-AS1* | -0.643 | 0.045 |
| *RNF145* | 0.612 | 0.049 | *UNC5B* | -0.643 | 0.039 |
| *VEGFB* | 0.612 | 0.040 | *RNF19B* | -0.643 | 0.039 |
| *PRDX1* | 0.611 | 0.045 | *ERI2* | -0.642 | 0.039 |
| *BMP4* | 0.610 | 0.054 | *C22orf46* | -0.641 | 0.043 |
| *OTUB2* | 0.609 | 0.058 | *ZNF563* | -0.638 | 0.032 |
| *LPCAT4* | 0.608 | 0.036 | *BSN* | -0.638 | 0.046 |
| *MSMO1* | 0.608 | 0.054 | *RHOU* | -0.636 | 0.023 |
| *FAM69A* | 0.607 | 0.058 | *SLC22A18* | -0.635 | 0.046 |
| *GJA1* | 0.607 | 0.058 | *AMZ2P1* | -0.635 | 0.044 |
| *NMT2* | 0.604 | 0.045 | *LOC100288842* | -0.633 | 0.039 |
| *FSCN1* | 0.603 | 0.044 | *PPP1R15A* | -0.633 | 0.049 |
| *LINC00707* | 0.602 | 0.046 | *CCDC171* | -0.633 | 0.046 |
| *USB1* | 0.598 | 0.031 | *SYBU* | -0.633 | 0.044 |
| *LSM3* | 0.598 | 0.059 | *GADD45A* | -0.632 | 0.038 |
| *MIR4435-1HG* | 0.598 | 0.061 | *WDR31* | -0.631 | 0.029 |
| *WDR83OS* | 0.597 | 0.058 | *YBEY* | -0.630 | 0.048 |
| *DNAJA1* | 0.597 | 0.052 | *ABLIM2* | -0.629 | 0.044 |
| *FAM89B* | 0.596 | 0.049 | *ANKMY1* | -0.628 | 0.046 |
| *VCL* | 0.595 | 0.059 | *PPP1R26-AS1* | -0.628 | 0.029 |
| *ISY1* | 0.595 | 0.058 | *CEBPD* | -0.627 | 0.049 |
| *PSMD6* | 0.594 | 0.048 | *SLC7A1* | -0.626 | 0.039 |
| *NAV1* | 0.593 | 0.061 | *LINC00909* | -0.626 | 0.051 |
| *MFSD5* | 0.593 | 0.055 | *LOC100506834* | -0.625 | 0.034 |
| *ERCC2* | 0.592 | 0.049 | *RBM43* | -0.625 | 0.043 |
| *PMEPA1* | 0.591 | 0.059 | *PDE4D* | -0.624 | 0.042 |
| *PIM2* | 0.591 | 0.053 | *DAPK2* | -0.622 | 0.024 |
| *PSMB2* | 0.591 | 0.040 | *PXN-AS1* | -0.622 | 0.042 |
| *CLDN11* | 0.590 | 0.065 | *ERMAP* | -0.620 | 0.053 |
| *ABHD17C* | 0.590 | 0.055 | *ZNF441* | -0.620 | 0.053 |
| *CITED2* | 0.589 | 0.058 | *TIGD2* | -0.620 | 0.054 |
| *SNHG16* | 0.588 | 0.051 | *SLC24A1* | -0.620 | 0.050 |
| *TET1* | 0.586 | 0.068 | *ZCWPW1* | -0.619 | 0.053 |
| *DANCR* | 0.585 | 0.047 | *C1S* | -0.617 | 0.055 |
| *MCAM* | 0.585 | 0.033 | *ZNF543* | -0.617 | 0.055 |
| *IL18* | 0.585 | 0.065 | *FUT1* | -0.616 | 0.045 |
|  |  |  | *RP9* | -0.615 | 0.051 |
|  |  |  | *HERPUD1* | -0.614 | 0.052 |
|  |  |  | *USP27X-AS1* | -0.612 | 0.038 |
|  |  |  | *APOL6* | -0.611 | 0.057 |
|  |  |  | *RSG1* | -0.611 | 0.055 |
|  |  |  | *N4BP2* | -0.611 | 0.056 |
|  |  |  | *PRIMPOL* | -0.610 | 0.058 |
|  |  |  | *ZBTB14* | -0.609 | 0.054 |
|  |  |  | *ZNF569* | -0.608 | 0.057 |
|  |  |  | *SCAND2P* | -0.608 | 0.051 |
|  |  |  | *STOX1* | -0.608 | 0.051 |
|  |  |  | *TIGD6* | -0.607 | 0.058 |
|  |  |  | *RFX3* | -0.607 | 0.052 |
|  |  |  | *ZBED3* | -0.606 | 0.059 |
|  |  |  | *SNHG8* | -0.606 | 0.059 |
|  |  |  | *JHDM1D-AS1* | -0.606 | 0.048 |
|  |  |  | *ZNF844* | -0.606 | 0.059 |
|  |  |  | *LYRM9* | -0.605 | 0.051 |
|  |  |  | *TTLL1* | -0.604 | 0.060 |
|  |  |  | *MXRA8* | -0.604 | 0.058 |
|  |  |  | *HKR1* | -0.603 | 0.058 |
|  |  |  | *AJUBA* | -0.603 | 0.040 |
|  |  |  | *LOC399815* | -0.602 | 0.061 |
|  |  |  | *PKD1* | -0.602 | 0.054 |
|  |  |  | *TCHP* | -0.602 | 0.051 |
|  |  |  | *DENND1B* | -0.601 | 0.060 |
|  |  |  | *LOC646762* | -0.601 | 0.061 |
|  |  |  | *SLC37A1* | -0.601 | 0.042 |
|  |  |  | *IRF1* | -0.600 | 0.058 |
|  |  |  | *SRP14-AS1* | -0.597 | 0.056 |
|  |  |  | *C2orf81* | -0.597 | 0.060 |
|  |  |  | *ZNF350* | -0.596 | 0.063 |
|  |  |  | *MPPE1* | -0.596 | 0.063 |
|  |  |  | *FBF1* | -0.596 | 0.053 |
|  |  |  | *LRRC37B* | -0.596 | 0.063 |
|  |  |  | *FAM86FP* | -0.594 | 0.063 |
|  |  |  | *PQLC3* | -0.594 | 0.063 |
|  |  |  | *RGS3* | -0.593 | 0.065 |
|  |  |  | *RFFL* | -0.591 | 0.066 |
|  |  |  | *BLZF1* | -0.591 | 0.063 |
|  |  |  | *SAYSD1* | -0.591 | 0.064 |
|  |  |  | *LONRF1* | -0.591 | 0.063 |
|  |  |  | *ITGA10* | -0.590 | 0.057 |
|  |  |  | *ABCA1* | -0.590 | 0.059 |
|  |  |  | *TRAM2-AS1* | -0.589 | 0.065 |
|  |  |  | *ZFP28* | -0.589 | 0.063 |
|  |  |  | *PTPN4* | -0.589 | 0.060 |
|  |  |  | *LETMD1* | -0.588 | 0.048 |
|  |  |  | *LRRC8B* | -0.588 | 0.067 |
|  |  |  | *STON2* | -0.588 | 0.063 |
|  |  |  | *DUSP8* | -0.588 | 0.063 |
|  |  |  | *PAPPA* | -0.588 | 0.065 |
|  |  |  | *TOP3A* | -0.587 | 0.046 |
|  |  |  | *LINC00346* | -0.586 | 0.046 |
|  |  |  | *ZNF426* | -0.586 | 0.064 |
|  |  |  | *P2RX6* | -0.586 | 0.057 |
|  |  |  | *ZSWIM3* | -0.585 | 0.064 |

Table I: Pathways identified through Sigora analysis of genes differentially expressed in cells that were pretreated with BPIFB1 prior to stimulation, compared to cells that were stimulated with *P. aeruginosa* alone

| **Pathway** | ***P* value (Unadjusted)** | ***P* value (Adjusted)** | **Signature Genes** |
| --- | --- | --- | --- |
| Gap junction trafficking and regulation | 6.51 × 10^-7^ | 5.72 × 10^-4^ | *AJUBA*  *FBF1*  *GJA1*  *SERPINE1*  *TIMP1*  *TUBA4A*  *VCL*  *VEGFA*  *VEGFB*  *VEGFC* |
| Platelet degranulation | 6.14 × 10^-6^ | 0.005 | *SERPINE1*  *TIMP1*  *VEGFA*  *VEGFB*  *VEGFC* |
| Metabolism of vitamins and cofactors | 1.97 × 10^-5^ | 0.017 | *GSTO1*  *MOCOS* |

Figure A**:** Gene expression in the IB3-1 CF airway epithelial cell line stimulated with heat-killed *P. aeruginosa* with and without pretreatment with recombinant BPIFA1 or BPIFB1 protein. N=1 for each condition. a) PCA plot generated using DESeq2 of whole transcriptome sequencing data from IB3-1 airway epithelial cells at baseline, stimulated with heat killed *P. aeruginosa*, pretreated with recombinant BPIFA1 or BPIFB1 prior to stimulation with heat killed *P. aeruginosa*, or treated with recombinant BPIFA1 or BPIFB1 alone b) heatmap of all genes that were differentially expressed in IB3-1 cells stimulated with *P. aeruginosa* compared to unstimulated cells.


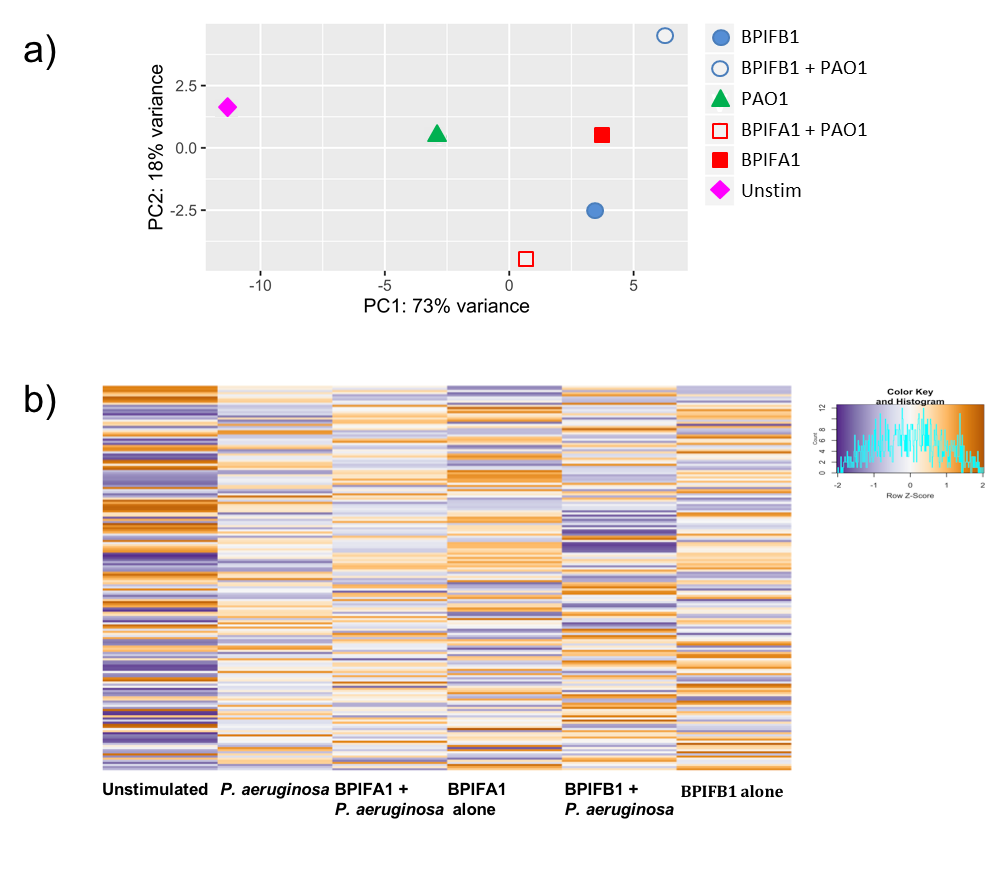


Figure B**:** Zero order protein-protein network of genes that were differentially expressed in response to BPIFA1 treatment in IB3-1 cells. Green color indicates genes that were upregulated in response to BPIFA1 and red indicates genes that were downregulated. The size of the node indicates the number of interaction partners. Proteins with many interaction partners (or hubs) are larger in size and are labeled with their name.


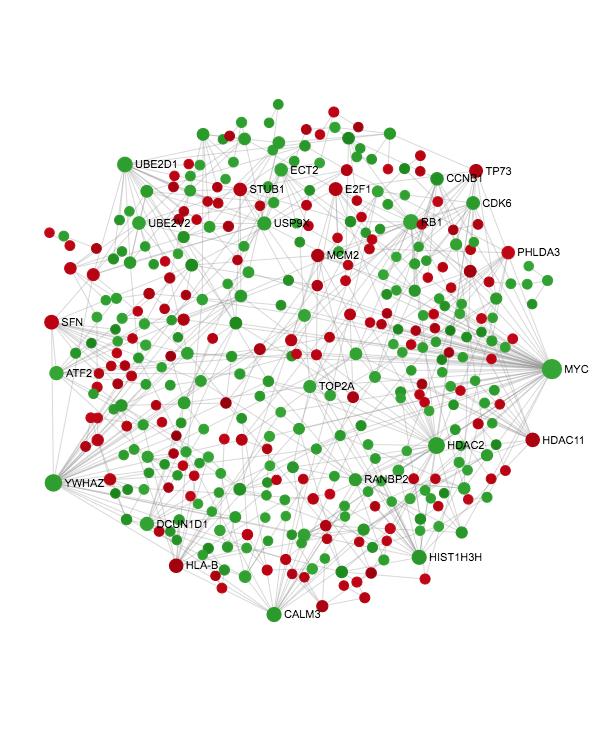


Figure C**:** Zero order protein-protein network of genes that were differentially expressed in response to BPIFB1 treatment in IB3-1 cells. Green color indicates genes that were upregulated in response to BPIFB1 and red indicates genes that were downregulated. The size of the node indicates the number of interaction partners. Proteins with many interaction partners (or hubs) are larger in size and are labeled with their name.


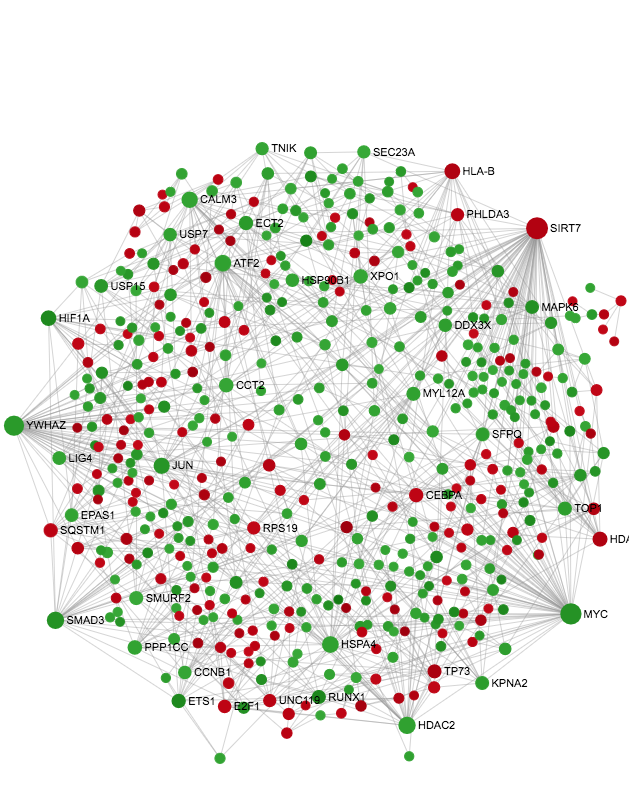


Figure D**:** First order protein-protein network of genes that were differentially expressed in response to BPIFA1 treatment in CFBE41o- cells. Green color indicates genes that were upregulated in response to BPIFA1 and red indicates genes that were downregulated. The size of the node indicates the number of interaction partners. Proteins with many interaction partners (or hubs) are larger in size and are labeled with their name.


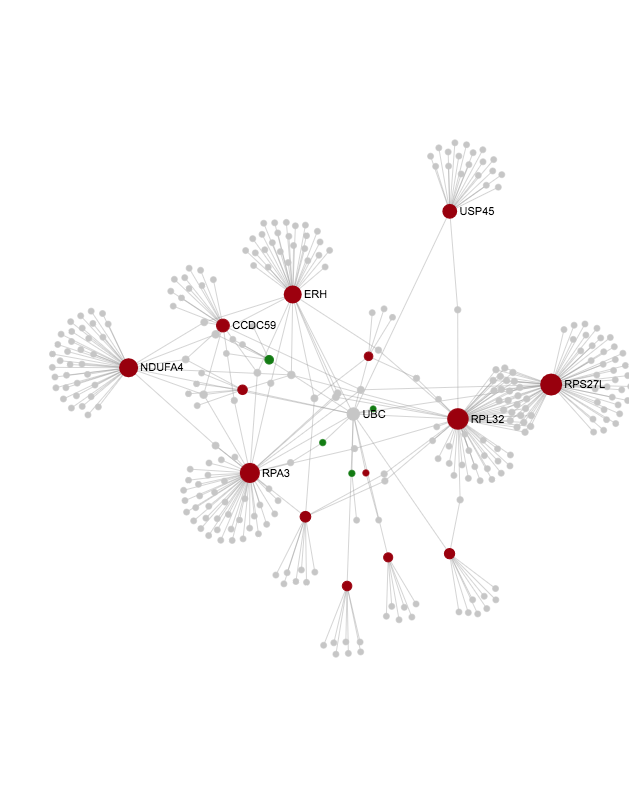


Figure E**:** First order protein-protein network of genes that were differentially expressed in response to BPIFB1 treatment in CFBE41o- cells. Green color indicates genes that were upregulated in response to BPIFB1 and red indicates genes that were downregulated. The size of the node indicates the number of interaction partners. Proteins with many interaction partners (or hubs) are larger in size and are labeled with their name.


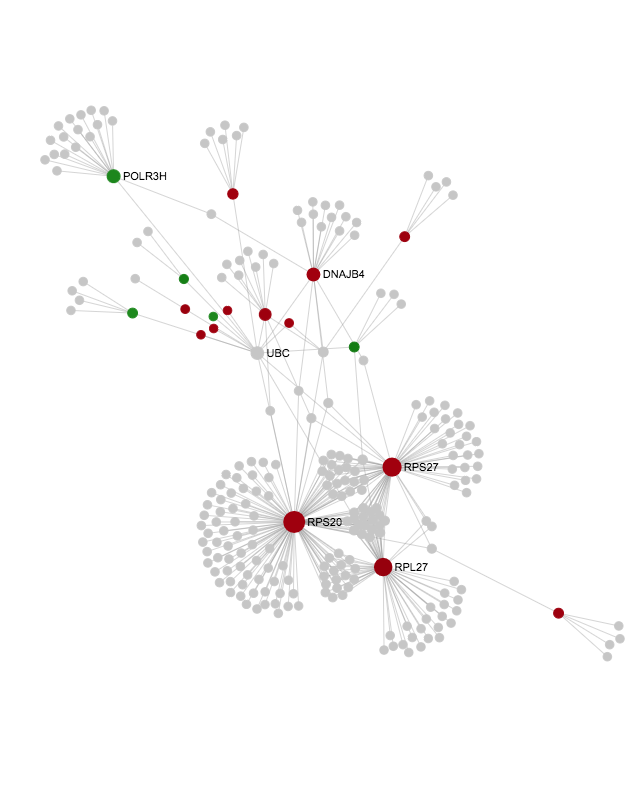


**REFERENCES**

1. Corvol H, Blackman SM, Boelle PY, Gallins PJ, Pace RG, Stonebraker JR, Accurso FJ, Clement A, Collaco JM, Dang H, Dang AT, Franca A, Gong J, Guillot L, Keenan K, Li W, Lin F, Patrone MV, Raraigh KS, Sun L, Zhou YH, O'Neal WK, Sontag MK, Levy H, Durie PR, Rommens JM, Drumm ML, Wright FA, Strug LJ, Cutting GR, Knowles MR. Genome-wide association meta-analysis identifies five modifier loci of lung disease severity in cystic fibrosis. Nat Commun 2015: 6: 8382.

2. Taylor C, Commander CW, Collaco JM, Strug LJ, Li W, Wright FA, Webel AD, Pace RG, Stonebraker JR, Naughton K, Dorfman R, Sandford A, Blackman SM, Berthiaume Y, Pare P, Drumm ML, Zielenski J, Durie P, Cutting GR, Knowles MR, Corey M. A novel lung disease phenotype adjusted for mortality attrition for cystic fibrosis Genetic modifier studies. Pediatr Pulmonol 2011: 46: 857-869.

3. Andrews S. FastQC: a quality control tool for high throughput sequence data. 2010: Available online at: https://[www.bioinformatics.babraham.ac.uk/projects/fastqc/](http://www.bioinformatics.babraham.ac.uk/projects/fastqc/).

4. Martin M. Cutadapt removes adapter sequences from high-throughput sequencing reads. EMBnetjournal 2011: 17: 10-12.

5. Kim D, Pertea G, Trapnell C, Pimentel H, Kelley R, Salzberg SL. TopHat2: accurate alignment of transcriptomes in the presence of insertions, deletions and gene fusions. Genome Biol 2013: 14: R36.

6. Langmead B, Salzberg SL. Fast gapped-read alignment with Bowtie 2. Nat Methods 2012: 9: 357-359.

7. Li H, Handsaker B, Wysoker A, Fennell T, Ruan J, Homer N, Marth G, Abecasis G, Durbin R, Genome Project Data Processing S. The Sequence Alignment/Map format and SAMtools. Bioinformatics 2009: 25: 2078-2079.

8. Love MI, Huber W, Anders S. Moderated estimation of fold change and dispersion for RNA-seq data with DESeq2. Genome Biol 2014: 15: 550.

9. Ritchie ME, Phipson B, Wu D, Hu Y, Law CW, Shi W, Smyth GK. limma powers differential expression analyses for RNA-sequencing and microarray studies. Nucleic Acids Res 2015: 43: e47.

10. Foroushani AB, Brinkman FS, Lynn DJ. Pathway-GPS and SIGORA: identifying relevant pathways based on the over-representation of their gene-pair signatures. PeerJ 2013: 1: e229.

11. Xia J, Gill EE, Hancock RE. NetworkAnalyst for statistical, visual and network-based meta-analysis of gene expression data. Nat Protoc 2015: 10: 823-844.
